# Epithelial cells sacrifice excess area to preserve fluidity in response to external mechanical stress

**DOI:** 10.1038/s42003-022-03809-8

**Published:** 2022-08-22

**Authors:** Jonathan F. E. Bodenschatz, Karim Ajmail, Mark Skamrahl, Marian Vache, Jannis Gottwald, Stefan Nehls, Andreas Janshoff

**Affiliations:** Georg-August Universität Göttingen, Institute of Physical Chemistry, Tammannstr. 6, 37077 Göttingen, Germany

**Keywords:** Nanoscale biophysics, Membrane biophysics

## Abstract

Viscoelastic properties of epithelial cells subject to shape changes were monitored by indentation-retraction/relaxation experiments. MDCK II cells cultured on extensible polydimethylsiloxane substrates were laterally stretched and, in response, displayed increased cortex contractility and loss of excess surface area. Thereby, the cells preserve their fluidity but inevitably become stiffer. We found similar behavior in demixed cell monolayers of ZO-1/2 double knock down (dKD) cells, cells exposed to different temperatures and after removal of cholesterol from the plasma membrane. Conversely, the mechanical response of single cells adhered onto differently sized patches displays no visible rheological change. Sacrificing excess surface area allows the cells to respond to mechanical challenges without losing their ability to flow. They gain a new degree of freedom that permits resolving the interdependence of fluidity *β* on stiffness ${K}_{{{{{{{{\rm{A}}}}}}}}}^{0}$. We also propose a model that permits to tell apart contributions from excess membrane area and excess cell surface area.

## Abstract (web summary)

The viscoelastic properties of cells subjected to external strain are assessed, showing that cells become stiffer but preserve fluidity by sacrificing their excess surface area.

## Introduction

Mammalian cells frequently experience substantial external tension that acts across the tissue, sometimes even facing cyclic stretch from the beating of the heart, pulsating blood vessels, or lung airways during breathing. The ability of cells to respond to these stimuli has important consequences for the organism.

Viscoelastic properties of cells play a pivotal role in all processes that involve substantial shape changes such as adhesion, spreading, growth, division, migration, carcinogenesis, and tissue development. It is widely accepted that the major response to external deformation originates from the actin cortex, a thin, reversibly cross-linked actin mesh that is either directly coupled to the plasma membrane through cross-linkers from the ezrin-radixin-moesin family or to the thin spectrin network that itself is anchored to the plasma membrane via ankyrins^1–7^. The membrane/cortex composite forms a dynamic shell that controls the cell’s shape and remodels on time scales of tens of seconds due to protein turnover and myosin-mediated contractions, thereby enabling cells to rapidly adapt and respond to external cues. A prominent example of a considerable shape change is the transition from rounded cells in suspension to spread, adherent ones^8–10^. As this transition occurs rapidly and under constant volume, there is insufficient time for substantial changes in surface area to occur, which requires the cell to rely on excess area stored in wrinkles, folds, caveolae, and other structures that exist on the cell’s surface. These structures are hardly visible in optical microscopy but have been recognized in high-resolution scanning/transmission electron micrographs and atomic force microscopy images^11–13^. It was shown that cells employ caveolae to buffer the variations of membrane tension induced by transient mechanical stress^14^. Also, in response to osmotic stress, cells recruit excess areas to protect themselves from lysis^13^. Kosmalska et al. showed that adherent cells passively remodel their membrane in response to changes in the area and volume of adherent cells prior to any active response^15^. This implies that cells react both passively on very short time scales and actively on longer time scales involving the cytoskeleton. Upon spreading, suspended cells release their excess surface area to avoid large stresses that might lead to cell wounding or lysis^10^. However, the ability of cells to quickly respond to changes in their environment by cytoskeletal remodeling is still not fully understood. For instance, it remains unclear whether excess surface area is limited to the plasma membrane or also includes a part of the cortex as a composite shell.

Here, we investigate how cells respond mechanically to external cues by directly targeting their excess surface area. For this purpose, we measure the viscoelastic properties of cells subject to external strain, varying spreading area, knock-down of tight junction proteins ZO-1/2, temperature change, and removal of cholesterol from the plasma membrane. The viscoelastic properties of the cortex include its prestress, which is predominately generated by the contractile properties of the reversibly cross-linked actomyosin network, the area compressibility modulus, which is essentially a two-dimensional elastic modulus of the composite shell and finally the fluidity of the network, i.e., the power-law exponent that characterizes the material within the extremes, a fluid (*β* = 1) or a solid (*β* = 0).

Previously, we and others found that cells display viscoelasticity that obeys a universal scaling behavior, in which fluidity, prestress, and stiffness are not independent variables^10,16–20^. Using the established approach to describe the cells as liquid-filled shells that maintain their volume upon deformation, we found that cells stiffen in response to the removal of excess area. Both the prestress and the area compressibility modulus increase with increasing area dilatation. To our surprise, however, all these processes maintain the fluidity of the cells. The cell’s fluidity, represented by the parameter *β*, reflects the energy dissipation upon deformation, as can be rationalized by the definition of the loss tangent^10^. In other words, *β* is the ratio of dissipated energy to elastically stored energy in the cortex shell. Therefore, cells change their viscoelastic properties along a trajectory, where *β* and ${K}_{{{{{{{{\rm{A}}}}}}}}}^{0}$ are not independent, as detailed previously^10,16,19^. We could also show that cell-like systems devoid of excess membrane area display extremely stiff cortices rendering both the abundance of surface area and the actomyosin contractility two important prerequisites for the cell to fulfill mechanically challenging tasks and to maintain mechanical homeostasis^10^.

The picture that emerges is that perturbing mechanical homeostasis on shorter time scales leads to a change in cellular stiffness, i.e., the area compressibility modulus, but not in fluidity (energy dissipation). This abolition of the dependency permits the cell to regain a degree of freedom to independently alter fluidity and stiffness by the amount of surface area. In contrast, if the cells were given enough time to adapt, e.g., if seeded on patterned surfaces of different sizes, homeostasis of cellular stiffness and fluidity is maintained.

## Results

### Cell stretching

**Fig. 1:**
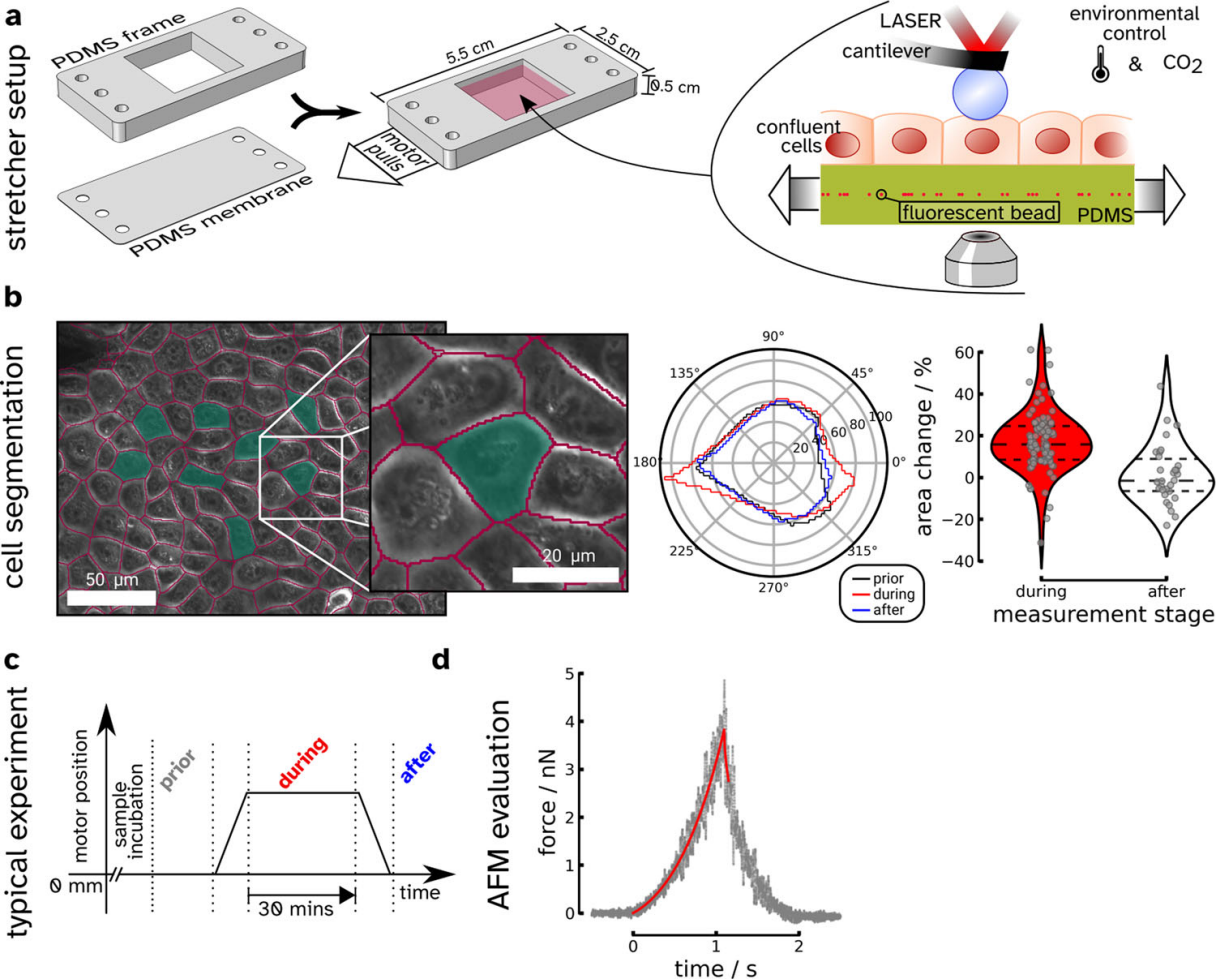
Overview of the cell stretcher experiment and the measured changes in cell properties due to strain. **a** Scheme illustrating the design and production of the polydimethylsiloxane (PDMS) measurement chamber and the final assembly. Cells are cultured to confluence and then strained along the longer axis of the chamber. The chamber is mounted on an optical microscope to perform AFM measurements under optical inspection and physiological cell culture conditions. **b** Left: example of cell segmentation of phase-contrast images carried out by Cellpose. Cell-cell contacts are marked in magenta, and the probed cells are colored in green. The change of the cell shape is followed over time during stretching. Right: change of the cell's projected area during (red) and after application of strain (white). Each point represents a single measured cell. The thick dashed line corresponds to the median, while the thin dashed lines refer to the upper and lower quartile, respectively (*n* = 90 cells during, *n* = 30 cells after). **c** Motor position and applied strain over the course of the experiment. **d** Typical indentation-retraction curve before the strain was applied (gray dots) together with the fit of equation (1) (red line, maximum indentation depth ${\delta }_{\max }=2.2\,\mu {{{{{{{\rm{m}}}}}}}}$, see Supplementary Fig. 8 for an overview of ${\delta }_{\max }$).

**Fig. 2:**
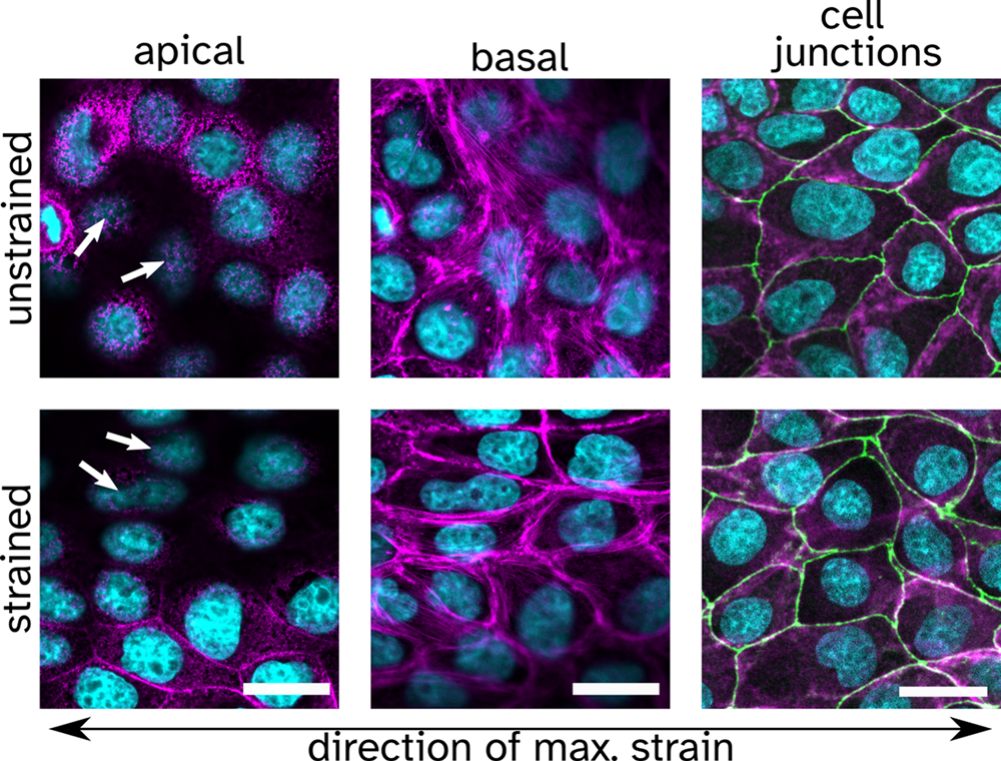
Immunostained confluent MDCK II cells showing the F-actin (Phalloidin 546) in magenta, the cell junction protein ZO1 in green (anti-ZO1 Alexa Fluor 488 monoclonal antibody), and the nucleus in cyan (DAPI). The apical and basal parts, as well as the cell junctions of the layer in a strained and unstrained state, are shown, respectively. The arrows point to locations in the unstrained and strained state where the microvilli's number and structure can be compared. Scale bar is 20 μm.

External stretching of a cell monolayer allows us to reversibly perturb the mechanical homeostasis of polarized epithelial cells and at the same time to measure the cellular response to this disturbance by time-resolved AFM (atomic force microscope) indentation-retraction experiments. Figure 1a shows the home-built setup used to perform uniaxial cell stretching experiments with confluent MDCK II cells cultured on polydimethylsiloxane (PDMS) substrates (see Supplementary Fig. 1/2 and Supplementary Note 1/2). Segmentation analysis prior, during, and after stretching provides the following cell shape parameters: cell area (apical), eccentricity, and orientation (see Supplementary Fig. 3 and Supplementary Note 3). All these properties were evaluated for each cell individually, and the changes were tracked throughout the experiment using the segmentation software Cellpose (see Fig. 1b)^21,22^. We found that an axial stretch of approximately 15 and 30% resulted in an average cellular area dilation of roughly 13 and 17%, respectively (see Fig. 1b and Supplementary Fig. 2/3). The orientation and eccentricity of the cell were inferred from an ellipse with the same second moments as given by the cell segmentation. The orientation, which is the angle of the major axis to the axis perpendicular to the axis of strain, shows an alignment with the strain direction. Eccentricity depends on the orientation before strain application because the PDMS membrane contracts in the lateral direction. When this is considered, cell eccentricity follows the expected orientation-dependent behavior (see Supplementary Fig. 3). All of the observed shape changes were fully reversible when external stress was removed. Images of the F-actin (apical and basal) cytoskeleton before and during applied external strain are shown in Fig. 2. On the apical side of the undisturbed cells, microvilli can be observed that vanish or spread out under the external strain. The reduction of microvilli indicates that, indeed, surface area reservoirs are removed upon stretching (see Supplementary Fig. 4). On the basal side of unstrained cells, the actin network shows no preferential orientation. However, after applying strain, the actin fibers run along the cell-cell walls and are preferentially oriented parallel to the strain direction.

Immunostaining of the tight junction protein ZO1 shows that also cell borders smooth out upon application of external strain, confirming that the strain exerted on the cells at the position of the focal adhesions is transferred to the top of the cells, essentially ironing out cell-cell boundaries. The originally “wrinkled” boundaries are now smoothed out by the strain, as shown in Fig. 2.

**Fig. 3:**
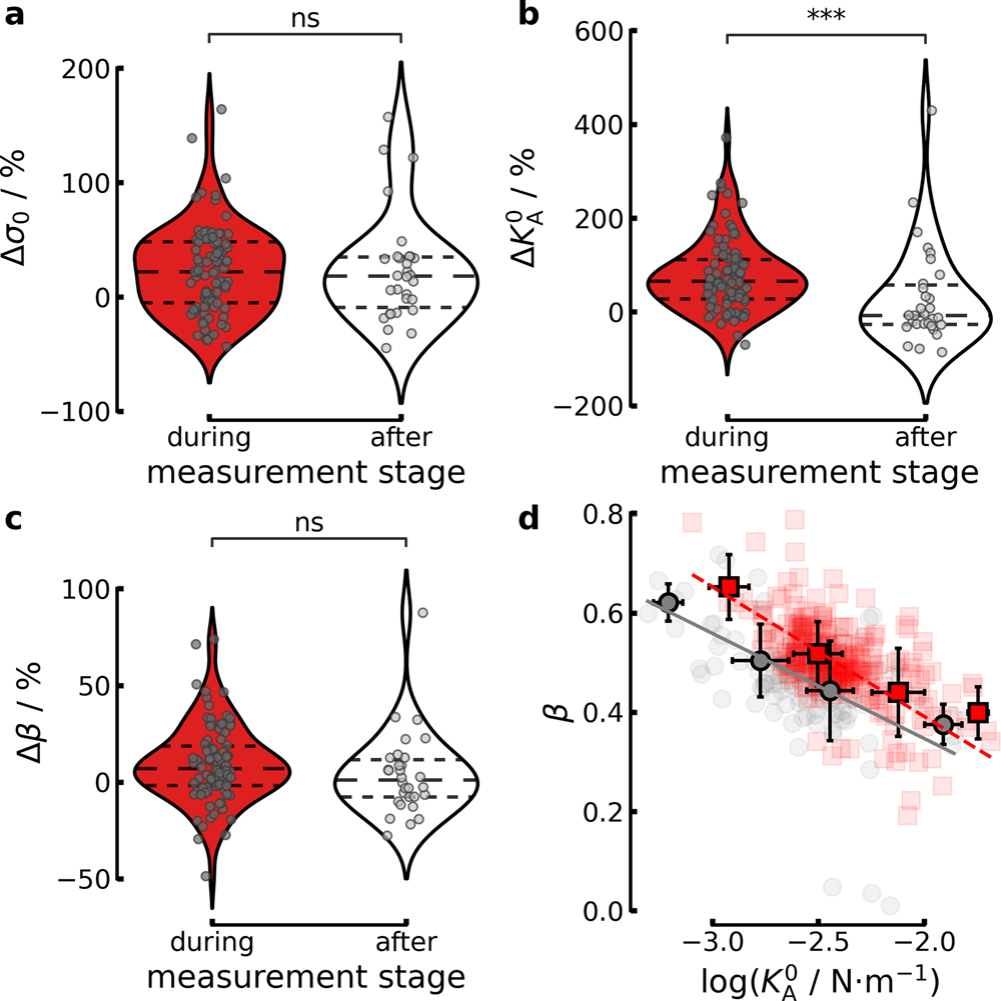
The changes in the mechanical properties of cells due to strain. For the maximum indentation depths *δ*_max_ see Supplementary Fig. 8. **a**–**c** Change of prestress Δ*σ*_0_, area compressibility modulus ${{\Delta }}{K}_{{{{{{{{\rm{A}}}}}}}}}^{0}$ and fluidity Δ*β* normalized to the values obtained prior to strain (in %). Red denotes the cells during strain and white corresponds to the cells after strain was released, respectively. The gray points are the changes of single cells averaged over multiple indentations and the dashed lines mark the quartiles. **d** Scaling of $\beta (\log ({K}_{{{{{{{{\rm{A}}}}}}}}}^{0}))$. Gray circles correspond to unstrained cells and red squares to the same cells after strain is applied, respectively (*n* = 90 cells during, *n* = 30 cells after). The dark gray circles (prior) and dark red squares (during) are the binned averages, with the error bars showing the standard deviation. The solid gray line and dashed red line is a linear fit of the averaged data, prior or during strain application, respectively.

To address the question of how external strain affects the viscoelastic properties, we performed indentation-relaxation experiments (force as a function of time) with colloidal probes prior, during, and after applying lateral strain (Fig. 3a/c) on single MDCK II cells of a confluent layer.

Previously, we showed that cell deformation experiments are well described by a viscoelastic cortex model comprising a pre-stressed capsule that obeys power-law rheology of the area compressibility modulus^16,19,20^. We assumed that the only source of resistance to deformation originates from the overall tension *σ* of the cell surface composed of *σ*_0_, the time-invariant prestress of the membrane/cortex shell and *K*_A_, the area compressibility modulus, which reflects the time-dependent elastic resistance to areal strain *α* = (Δ*A*/*A*_0_)^23^. Viscoelasticity of this 2D elastic modulus is assumed to enter through a power law ${K}_{{{{{{{{\rm{A}}}}}}}}}={K}_{{{{{{{{\rm{A}}}}}}}}}^{0}{\left(\frac{t}{{t}_{0}}\right)}^{-\beta }$ with 0 ≤ *β* ≤ 1 and *t*_0_ = 1 s (set arbitrarily)^19^. Application of the elastic-viscoelastic-correspondence principle^24–26^ leads to the following expression for the force response:

1 $$f=2\pi {\widetilde{g}}_{{{{{{{{\rm{cap}}}}}}}}}(\widetilde{z}){R}_{1}\left({\sigma }_{0}+\int\nolimits_{0}^{t}{K}_{{{{{{{{\rm{A}}}}}}}}}^{0}{\left(\frac{t-\tau }{{t}_{0}}\right)}^{-\beta }\frac{\partial \alpha (\tau )}{\partial \tau }{{{{{{{\rm{d}}}}}}}}\tau \right).$$

${\widetilde{g}}_{{{{{{{{\rm{cap}}}}}}}}}$ is the generic shape function for a given indentation depth $\widetilde{z}=z/{R}_{1}$ and *R*_1_ the radius of the apical cap (see Supplementary Note 5 for details)^10^. A typical force curve resulting from the indentation-retraction experiment is depicted in Fig. 1d. Notably, the thin PDMS membrane frequently generates unwanted oscillations in AFM force curves compared to cells cultured on Petri dishes which results in lower true indentation forces^27^. The red line represents the outcome of the fit according to equation (1), employing a piecewise function for indentation and retraction/relaxation, respectively (see Supplementary Note 5 for details).

Generally, three independent parameters are obtained, the prestress of the cortex *σ*_0_, the scaling parameter ${K}_{{{{{{{{\rm{A}}}}}}}}}^{0}$ and the fluidity or power-law exponent *β*. In most cases, only the initial part of the retraction curve was included in the fitted data to minimize perturbation from adhesion effects^19^.

In response to external strain, the prestress *σ*_0_ increases by ~20%. Unexpectedly, this increase in tension persists even after the strain is released (Fig. 3a), indicating that the active cortex response is long-lived (>30 min) in contrast to passive relaxation processes decaying on the order of seconds (vide infra). The scaling factor ${K}_{{{{{{{{\rm{A}}}}}}}}}^{0}$, representing the stiffness of the cell, increases substantially by more than 60% (Fig. 3b), while the fluidity *β* changes only marginally (Fig. 3c). It is important to note that, unlike the prestress *σ*_0_, ${K}_{{{{{{{{\rm{A}}}}}}}}}^{0}$ and *β* assume their original values after the lateral stress is removed and the cell shape restored (Fig. 3b/c). Additionally, then values remain constant over the 30 min strain step (see Supplementary Fig. 5).

This finding can be explained by the presence of excess surface area, which passively contributes to the two-dimensional elasticity represented by the area compressibility modulus *K*_A_. The apparent area compressibility modulus generated by the cell is reduced by an excess surface that can be sacrificed to compensate for the build-up of lateral stress. While both cortical tension *σ*_0_ and fluidity *β* are the result of motor proteins consuming chemical energy, the loss or restoration of the excess surface area is considered a largely passive process. Exceptions are active processes involving endocytosis and exocytosis of vesicles. As explained below, cortical tension acts in the opposite direction to external tension, promoting the reversible formation of excess area stored in folds and wrinkles. These structures can release the trapped area during lateral dilation and intercept its consequences^28^.

Recently, we could show that cellular fluidity represented by the power-law exponent *β* and the scaling factor ${K}_{{{{{{{{\rm{A}}}}}}}}}^{0}$ are not independent^10,19^. We found that $\beta \propto -\log ({K}_{{{{{{{{\rm{A}}}}}}}}}^{0})$ (Fig. 3d) and similar correlations between cell stiffness and fluidity were reported by others^16^. This relationship implies that cells can only change fluidity and stiffness along a special trajectory. Surprisingly, however, after applying an external strain, we found that dilated cells show an almost parallel shift of the linear relationship $\beta (\log ({K}_{{{{{{{{\rm{A}}}}}}}}}^{0}))$ towards larger stiffness. This implies that while fluidity is preserved, stiffness increases independently upon dilatation, which we attribute to partial exhaustion of surface area reservoirs stored in ruffles, wrinkles, folds, microvilli, vesicles, and caveolae.

### Removal of surface reservoirs by methyl-*β*-cyclodextrin

**Fig. 4:**
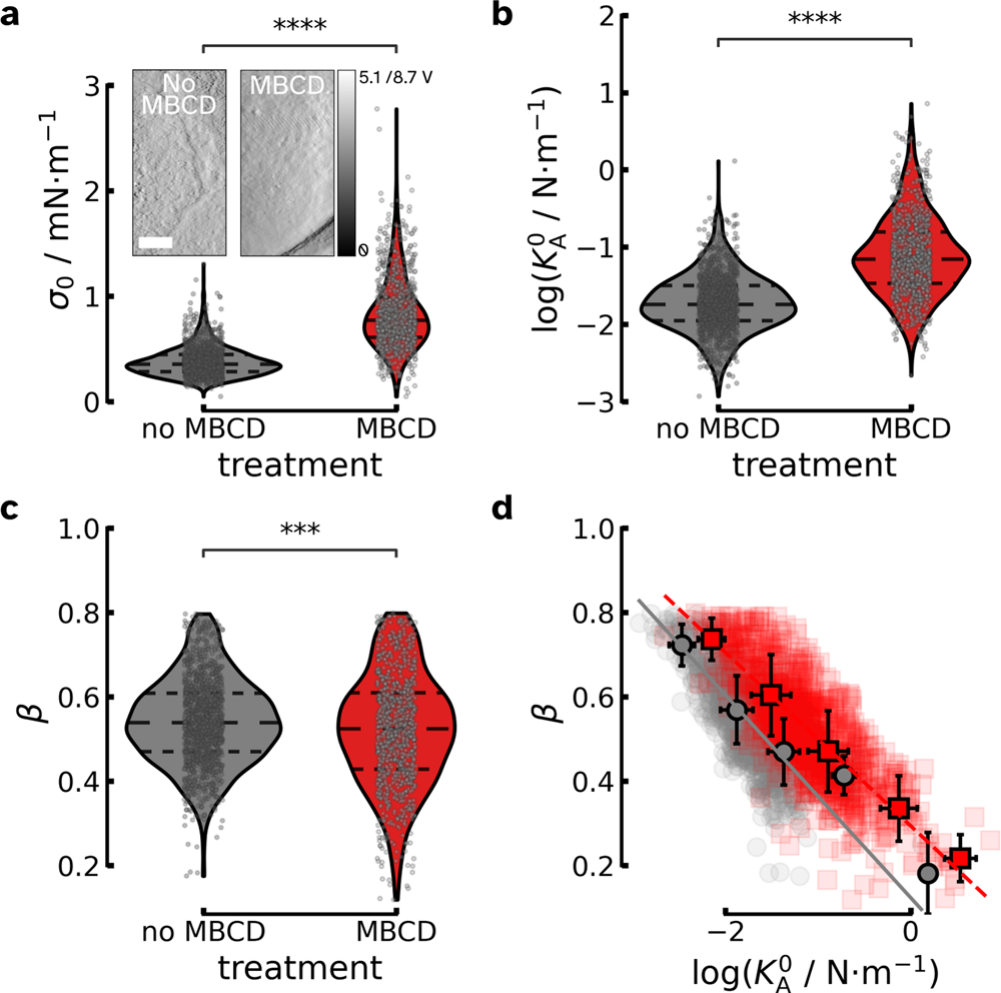
Methyl-*β* cyclodextrin (MBCD) treatment of MDCK II cells alters their viscoelastic response. For the maximum indentation depths *δ*_max_ see Supplementary Fig. 8. **a**–**c** Prestress/cortical tension *σ*_0_ and area compressibility modulus ${K}_{{{{{{{{\rm{A}}}}}}}}}^{0}$ increases after MBCD incubation (red), while the fluidity *β* remains constant. Each point represents the analysis of a single indentation-retraction measurement (no MBCD *n* = 1238 curves (10 cells); with MBCD *n* = 961 curves (10 cells) including also data from Pietuch et al. reevaluated with equation (1)^13,65^. **a** The inset shows contact mode vertical deflection images of fixated cells before and after exposure to MBCD. **d** Fluidity *β* depends linearly on the logarithm of the area compressibility modulus ${K}_{{{{{{{{\rm{A}}}}}}}}}^{0}$. The individual measurements are binned in two dimensions, means are shown. Untreated MDCK II cells are shown in gray and MBCD-treated ones are colored in red, respectively. The solid gray line represents a linear fit to the averaged data of untreated cells, while the dashed red line shows the linear fit to the averaged data of MBCD-treated cells.

An elegant way to reversibly clear the cell’s apical surface from features such as microvilli was introduced by Poole et al. using methyl-*β* cyclodextrin (MBCD) to remove preferentially cholesterol from the plasma membrane^12^. The same protocol was previously also used by our group to show that the elastic properties of cells are heavily impacted by the reversible removal of cholesterol from the plasma membrane^13^. Here, we focused on the viscoelastic response of confluent MDCK II cells to cholesterol extraction and removal of surface reservoirs from the apical side (Fig. 4). We observed that the prestress *σ*_0_ increases (Fig. 4a) and similar to the area dilation by application of external strain, MBCD administration leads to a parallel shift of the $\beta (\log ({K}_{{{{{{{{\rm{A}}}}}}}}}^{0}))$ relationship to higher cellular stiffness (Fig. 4d). We interpret this in terms of the cells sacrificing excess surface area but preserving the inherent mechanical properties of the cortex. This view is supported by experiments in which we lowered the temperature to stall active processes involved in area regulation leading to a stiffening of the cells (see Supplementary Fig. 6 and Supplementary Note 4). Generally, an increase in cortex tension prevents the outstretched plasma membrane from lysis, as also shown in the previous experiment. This combined behavior permits the cells to respond quickly to mechanical challenges such as osmotic shocks or external deformation without giving up the mechanical homeostasis of the cortex and avoiding damage.

### Stretching of cells within a demixed cell monolayer

**Fig. 5:**
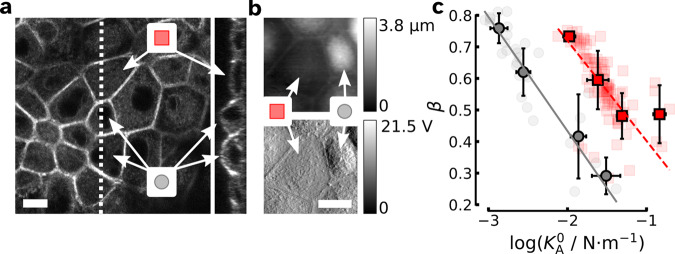
Internal cell stress generated by adjacent ZO1/2 dKD MDCK II cells. **a** Confocal laser scanning image (CLSM, F-actin stained) of typical cells chosen for indentation-relaxation experiments. A corresponding *yz*-slice of the image stack at the indicated position (dashed white line) is shown on the right. Small cells are marked with a gray circle, while large cells are represented by a red square. Scale bar is 10 μm. **b** AFM contact mode topography images of stretched and highly contractile cells. Measured height on top and the error signal is below. Scale bar is 10 μm. **c** Small (gray) and large (red) ZO1/2-depleted MDCK II cells subject to indentation-relaxation display distinctly different $\beta (\log ({K}_{{{{{{{{\rm{A}}}}}}}}}^{0}))$ trends. Mechanical data is taken from Skamrahl et al.^29^. While smaller, contractile cells are extremely soft (gray circles, continuous line, *n* = 30 cells), the larger and outstretched MDCK II cells (red squares, dashed line, *n* = 93 cells) are almost an order of magnitude stiffer.

Previously, we investigated the tug-of-war taking place in ZO1/2 dKD MDCK II cell monolayers due to the emergence of two coexisting species, small, highly contractile cells and stretched-out, elongated cells^29^. We found that the smaller cells exert considerable forces on their larger neighbors and are substantially higher than the larger but flatter counterparts due to volume conversation. The apical surface of the smaller cells displays a perijunctional contractile actin ring and large quantities of ruffled excess surface area, i.e., plasma membrane with attached cortex, is visible in confocal stack images and topographic images (Fig. 5a, b). Mechanical properties assessed by indentation-relaxation experiments revealed that prestress is increased on the larger outstretched cells (see Supplementary Fig. 7a, reproduced from Skamrahl et al.^29^), which at the same time display a substantial shift of the straight $\beta (\log ({K}_{{{{{{{{\rm{A}}}}}}}}}^{0}))$ lines towards larger cell stiffness ${K}_{{{{{{{{\rm{A}}}}}}}}}^{0}$ (Fig. 5c). For this purpose, we have re-plotted the data from Skamrahl et al.^29^. The difference in viscoelasticity between the small and large cells is therefore mainly in contractility and excess surface area. This means that cells respond to immediate external stress by increasing internal contractility and at the same time sacrificing excess surface area to mitigate the external stress. The two effects go hand in hand since higher cortex contractility fosters the generation of the excess plasma membrane, effectively reducing its in-plane tension^28^. But what happens if the cells are given enough time to adapt to the mechanical challenge? To rule out the possibility that cell size alone was responsible for the findings, we performed indentation-relaxation experiments with cells of different sizes using micro-patterns of extracellular matrix proteins to predefine cell spreading area^30^.

### Single cells cultured on differently-sized ECM footprints

**Fig. 6:**
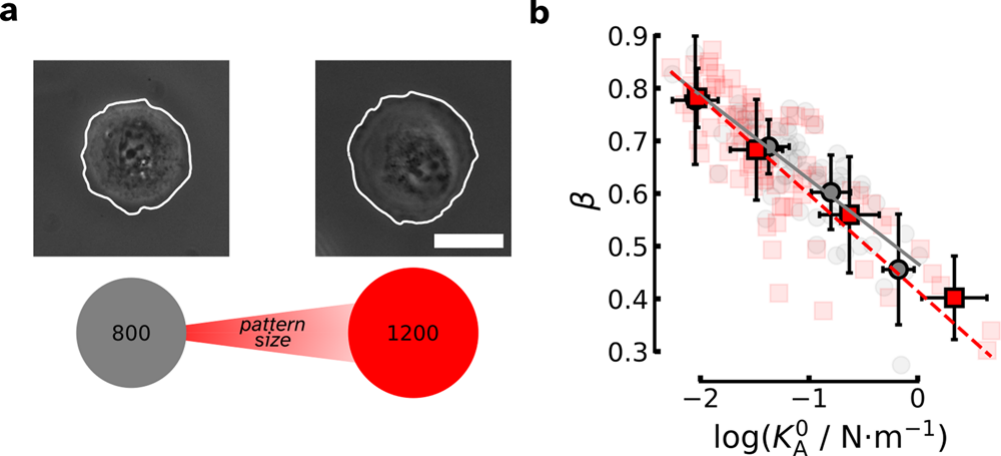
Impact of stress generated by different extracellular matrix (ECM) sizes on cellular mechanics. **a** Phase-contrast micrographs of single MDCK II cells (outlined in white) adhered to ECM spots with different sizes, 800 μm^2^ on the left and 1200 μm^2^ on the right. Scale bar is 20 μm. **b** Impact of cell size on the $\beta (\log ({K}_{{{{{{{{\rm{A}}}}}}}}}^{0}))$ scaling. The gray circles denote MDCK II cells adhered to the 800 μm^2^ large extracellular matrix (ECM) spot size with a gray solid trend line (*n* = 53 curves, 12 cells). The red squares correspond to cells cultured on a 1200 μm^2^ large ECM spot with a dashed red line indicating the linear trend (*n* = 77 curves, 15 cells.) For the maximum indentation depths, *δ*_max_ see Supplementary Fig. 8^30^.

We cultivated single MDCK II cells on circular extracellular matrix (ECM) spots of different sizes (see Fig. 6a) ranging from 800 to 1200 μm^2^ ^30^. If cells are unable to adjust their surface area by producing more membranes and cytoskeletal filaments, we expected a shift in the linear $\beta (\log ({K}_{{{{{{{{\rm{A}}}}}}}}}^{0}))$ relationship to higher cell stiffness as the patch size increased. However, our data for different spot sizes clearly show that this is not the case. No visible difference was observed between the $\beta (\log ({K}_{{{{{{{{\rm{A}}}}}}}}}^{0}))$ lines (Fig. 6b and Supplementary Fig. 7b) of cells attached to different spot sizes. This suggests that cells cultured on larger spots generate a sufficient amount of cell surface material to maintain mechanical homeostasis in response to the larger available area. However, this process stops when maximum size is reached, which is typically occupied by single adherent cells. This result differs from studies in which cells are cultured on soft gels or porous surfaces, where surface quality and not spot size affect their mechanical properties^31,32^.

### Giant liposomes filled with actin

**Fig. 7:**
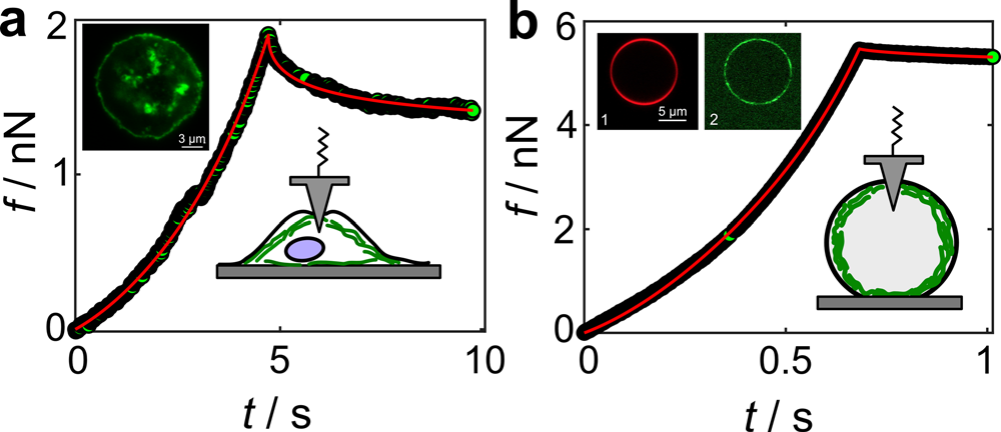
Giant liposomes versus epithelial cells. Comparison of force-relaxation experiments performed on individual epithelial cells (**a**) and actin-filled giant liposomes (**b**). The insets show representative confocal images of the cells and giant liposomes, with the actin cytoskeleton labeled in green (Alexa Fluor 488 actin) and the plasma membrane in red (Texas Red DHPE), respectively. Fits with the corresponding shape function to the force-relaxation data are shown as red lines corresponding to *σ*_0_ = 0.5 mN m^−1^, ${K}_{{{{{{{{\rm{A}}}}}}}}}^{0}=0.05$ N m^−1^ and *β* = 0.6 for the MDCK II cell (${\delta }_{\max }=2.5\,\mu {{{{{{{\rm{m}}}}}}}}$, *v*_0_ = 0.5 μm/s, *R*_cell_ = 12 μm) and *σ*_0_ = 2.7 mN m^−1^, ${K}_{{{{{{{{\rm{A}}}}}}}}}^{0}=0.11$ N m^−1^ and *β* = 0.07 for the GUV (${\delta }_{\max }=1.6\,\mu {{{{{{{\rm{m}}}}}}}}$, *v*_0_ = 2.7 μm/s, *R*_vesicle_ = 8.5 μm).

A paradigm for a cell lacking any excess surface reservoirs is an adhered giant unilamellar vesicle (GUV). Adhesion to the substrate can be very strong and thereby imposes considerable tension (prestress) on the shell ironing out most of the undulations that would be a source of recruitable excess area^33^. Here, we compare the viscoelastic response of a spread MDCK II cell with that of an actin-filled GUV mimicking the actin cortex (see Fig. 7). The GUV provides a limiting case with no surface reservoirs and a potentially dominating response from the membrane as the bilayer is largely inextensible as opposed to the compliant actin mesh^34,35^.

While the force relaxation (here at the constant *z*-piezo position, i.e., *v*_0_ = 0) on a single adherent living cell displays a fast decay indicating a typical softness with large *β* values and a substantial relaxation of stress, the actin-coated GUV remains solid-like with very small *β*-values, close to zero. This almost fully elastic system exhibits an area compressibility modulus close to that of a pure membrane (*K*_A_ ≈ 0.2 N m^−1^)^34^.

## Discussion

We found that confluent epithelial cells alter their mechanical properties in response to external stimuli targeting specifically the amount of available surface area. Applying lateral strain to the cells within the monolayer either externally or internally, reducing the temperature of the culture medium (see Supplementary Fig. 6 and Supplementary Note 4), or removing cholesterol from the plasma membrane all led to a stiffening of cells independently of cellular fluidity. Concomitantly, cortical tension (*σ*_0_) increases moderately except for the case where the temperature of the culture medium is decreased, putting the cortex into the rigor state (see Supplementary Fig. 6a). In rigor, all cross-links remain attached to actin, but they are not sliding. Overall, this observation is highly surprising since most mechanical triggers such as the addition of various cytoskeletal drugs such as latrunculin A, blebbistatin, jasplakinolide, or cytochalasin, the addition of cross-linkers such as glutardialdehyde and alteration of substrate properties usually change viscoelastic properties of cells along a specific trajectory. It was found before that stiffness (Young’s modulus *E*, shear modulus *G*, or area compressibility modulus *K*_A_) and fluidity (*β*) cannot be changed independently^16–19,36,37^. This view is largely explainable by envisioning the cytoskeleton to constitute a conformational energy landscape that displays many local minima with high energy barriers serving as kinetic traps similar to a soft glassy material^38^. If the network is deformed elastically, its filaments must remain in the energy wells, but once a filament escapes its trap by, for instance, myosin activity, its elastically stored deformation energy is dissipated as heat^17^. This implies a direct correlation between power loss and elasticity, leaving only one degree of freedom for the cell to choose in order to respond to external and internal stimuli. For instance, in order to alter ${K}_{{{{{{{{\rm{A}}}}}}}}}^{0}$, the cell only needs to change fluidity and vice versa.

Generally, the area compressibility modulus, as inferred from the F-actin mesh size (≈50 nm), thickness (≈100 nm), and persistence length (≈15 μm) is very small, on the order of 0.1–1 mN m^−1^ ^39^. Compared to a lipid bilayer that displays a modulus on the order of 0.1 N m^−1^ this would be a negligible contribution to the overall compressibility^23,34^. The presence of the plasma membrane, however, not only imposes an entropy penalty and thereby substantially stiffens the fluctuating network but also provides a large amount of linker sites that act essentially as cross-linkers, further solidifying the cortex by restricting the motion of the filaments^40^. Boey et al. investigated a polymer chain model for the human erythrocyte cytoskeleton and found that the area compressibility modulus increases with external stress^41^. This is unexpected from simple model systems composed of springs but a result of the reduced network fluctuations at larger dilatation^42^. This increase in the area compressibility modulus as a result of dilatation and suppression of motion is, however, unlikely to provide the explanation for the observed shift of $\beta (\log ({K}_{{{{{{{{\rm{A}}}}}}}}}^{0}))$ lines to higher stiffness values. A stretch-induced cell stiffening would inevitably reduce dissipation, i.e., generate smaller values for *β*. This was, however, not observed. Conversely, only an increase in stiffness was measured, with negligible change in energy dissipation. The only conceivable explanation for this phenomenon is a reduction of excess overall surface area.

**Fig. 8:**
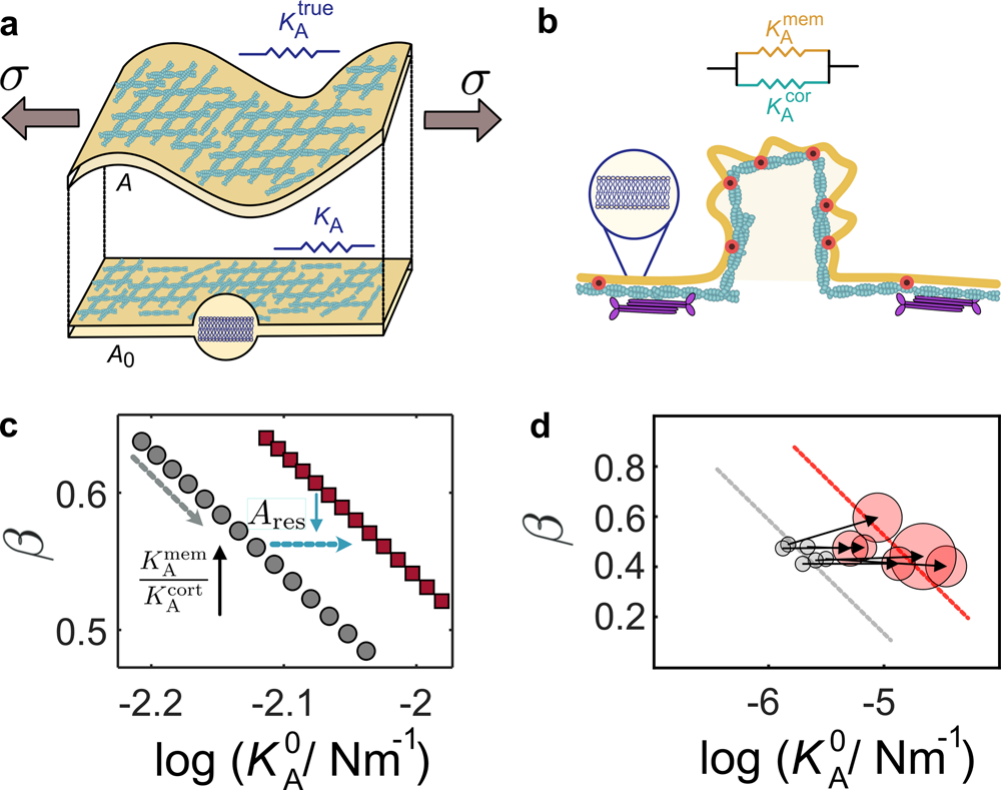
The impact of excess surface area and excess membrane on the (apparent) area compressibility modulus and viscoelastic response. **a** Definition of the apparent area compressibility modulus *K*_A_ referring to the projected area *A*_0_ of the true area *A* on the *xy* plane. **b** Neat membrane reservoirs and coexisting cortex-lined surface reservoirs. **c** Apparent fluidity $\widetilde{\beta }$ as a function of scaling factor ${K}_{{{{{{{{\rm{A}}}}}}}}}^{0}$ obtained from fits of equation (1) to data computed from equation (3). A linear decrease in apparent fluidity (gray dotted arrow) is found when excess membrane area is removed (*σ*_0_ = 0.3 mN m^−1^, ${K}_{{{{{{{{\rm{A}}}}}}}}}^{{{{{{{{\rm{cort}}}}}}}}}=0.006$ N m^−1^, *β* = 0.65, *R* = 10 μm, *v* = 1 μm s^−1^). ${K}_{{{{{{{{\rm{A}}}}}}}}}^{{{{{{{{\rm{mem}}}}}}}}}$ increases from 0.005 N m^−1^ (high excess area) to 0.25 N m^−1^ (absence of membrane reservoirs), leading to fitting results that misinterpret this as cortical stiffening. Also, a parallel shift of $\widetilde{\beta }\propto -\log ({K}_{{{{{{{{\rm{A}}}}}}}}}^{0})$ to stiffer cells can be created when the overall excess surface area is removed by 25% (blue dotted arrow). **d** Mechanical properties of individual cells before (gray circles) and after stretching (red circles). The size of the circles indicates the change in the area. Each individual cell exhibits a shift to larger stiffness (black arrows). The dotted lines are linear approximations to guide the eye.

Figure 8 illustrates the projection of the full area of the cortex *A* on *A*_0_ leading to apparent values for the area compressibility modulus, which is the experimentally accessible parameter (*K*_A_). In contrast, we denote the “true” surface compressibility modulus, i.e., that which a cortex would exhibit if it expanded laterally, as ${K}_{{{{{{{{\rm{A}}}}}}}}}^{{{{{{{{\rm{true}}}}}}}}}=A{\left(\frac{\partial \sigma }{\partial A}\right)}_{{{{{{{{\rm{V,T}}}}}}}}}$, and the contribution that comes exclusively from excess area or reservoirs as ${K}_{{{{{{{{\rm{A}}}}}}}}}^{{{{{{{{\rm{res}}}}}}}}}$. Surface reservoirs may comprise thermally excited undulations and more permanent structures depending on cell type or polarity. The two contributions, extensibility of the cortex and recruitment of excess surface area, resist area dilatation as two 2D “springs” arranged in series (see Fig. 8a), leading to an apparent area compressibility modulus related to the projected area *A*_0_ (see Supplementary Note 6)^43^:

2 $${K}_{{{{{{{{\rm{A}}}}}}}}}={A}_{0}{\left(\frac{\partial \sigma }{\partial {A}_{0}}\right)}_{V,T}=\frac{{A}_{0}}{A}\frac{{K}_{{{{{{{{\rm{A}}}}}}}}}^{{{{{{{{\rm{true}}}}}}}}}{K}_{{{{{{{{\rm{A}}}}}}}}}^{{{{{{{{\rm{res}}}}}}}}}}{{K}_{{{{{{{{\rm{A}}}}}}}}}^{{{{{{{{\rm{true}}}}}}}}}+{K}_{{{{{{{{\rm{A}}}}}}}}}^{{{{{{{{\rm{res}}}}}}}}}}=\xi {K}_{{{{{{{{\rm{A}}}}}}}}}^{{{{{{{{\rm{eff}}}}}}}}}.$$

Here, the excess area enters through a factor of $\xi =\frac{{A}_{0}}{{A}_{0}+{A}_{{{{{{{{\rm{res}}}}}}}}}}$ into the apparent modulus *K*_A_ and thereby modulates the response of cells to deformations. A large amount of excess surface area clearly softens the cell, while exhaustion of surface area reservoirs leads to stiffening and might eventually reveal the “true” viscoelastic properties of the cortex/membrane shell in the absence of excess area. The nearly parallel shift of $\beta (\log ({K}_{{{{{{{{\rm{A}}}}}}}}}^{0}))$ to larger apparent stiffness (${K}_{{{{{{{{\rm{A}}}}}}}}}^{0}$) can be expressed in terms of reduction of surface area by a factor of *ξ*^−1^. *ξ*^−1^ ranges from 1.5 through external stretching of the cell monolayer up to 6 for dkD MDCK II cells (small contractile species with large visible surface reservoirs), respectively (see Figs. 3d, 5c).

If we now revisit our data from stretching experiments and focus on the mechanical fate of individual MDCK II cells, we find that an increase in the cell area causes indeed a parallel shift to higher stiffness, i.e., larger ${K}_{{{{{{{{\rm{A}}}}}}}}}^{0}$ values at no change in fluidity (Fig. 8d). Figure 8d exemplarily shows the trajectory (arrows) for individual cells upon external dilatation. The spot-size of the red circles indicates the estimated area change upon stretching. The observed cell stiffening could, in principle, be explained by an inherent change of the area compressibility under external lateral stress.

Previously, we investigated the impact of substrate porosity on the mechanical properties of MDCK II cells^32^. Interestingly, the fluidity also remained unchanged, while the cells became smaller with a higher corrugated surfaces at constant volume and concomitantly also softer on substrates with larger pores. There also, the excess area of cells on large pores was responsible for the apparent softening of the cortex compared with more outstretched cells.

Taken together, the measured, apparent area compressibility modulus includes the impact of reservoir recruitment through an effective two-dimensional spring and softens through the presence of excess area. Obviously, this effect is only a geometric feature of the cell’s surface and not an intrinsic mechanical property of the pre-stressed network as it would be in the absence of reservoirs. Employing the strategy of sacrificing surface area reservoirs permits the cell to increase the apparent stiffness without changing the inherent energy dissipation of the cytoskeleton represented by the fluidity parameter *β*. This route offers the cells a quick protective response to external deformation by stiffening their cortex without sacrificing their ability to flow.

This concept only works as long as the membrane itself provides additional reservoirs to allow this kind of modulation over a wider range. The lipid bilayer is an almost inextensible liquid crystal that exhibits an area compressibility modulus on the order of 0.1 to 1 N m^−1^ depending on the lipid and protein composition^34^. Therefore, the neat plasma membrane might even dominate the mechanical response in the absence of any reservoirs, as illustrated by the experiment shown in Fig. 7. Pre-stressed giant liposomes display values of 0.2–0.4 N m^−1^ in AFM indention experiments being at the upper limit of values found for epithelial cells^34,44^. The limited extensibility of the lipid bilayer also poses a threat of possible cell lysis since about 3–5% of area expansion already leads to rupture of the plasma membrane.

Therefore, the cells need to stock up excess membrane area beyond that of the cortex to mitigate the impact of the membrane on the mechanical response. Usually, this requires local decoupling of the plasma membrane from the underlying cortex^13,15,45^. In order to recruit excess membrane in microvilli, the actin cytoskeleton needs to undergo continuous remodeling^45^. It was shown that F-actin continually forms excess membrane reservoirs up into dynamic microvilli to create a transient and highly plastic storage compartment^45^. Plasma membrane vesiculation through exocytosis to restore membrane tension and vesicle recruitment (endocytosis) to relieve tension are well-known to occur in a variety of cell types. Besides, membrane blebs have been shown to provide important reservoirs and functions for motility and apoptosis^46,47^. Other sources of excess membrane material might originate from microvilli, caveolae, or internal vesicles^48–50^. Gauthier et al. investigated the spreading dynamics of fibroblast, and since during this process, the plasma membrane area cannot increase by stretch, spreading proceeds by the flattening of membrane folds and/or by the addition of new membrane^51^. Using optical tweezers, they found that membrane tension progressively decreases during spreading, suggesting the addition of a new membrane through vesicle fusion. They observed that internal membrane structures are sacrificed to provide vesicles for exocytosis during spreading. In a follow-up study, Gauthier and coworkers also showed that membrane area and tension are important regulators of a fundamental immune response^52^. Other cells respond to external deformation in two subsequent phases, starting with initial plasma membrane unfolding, followed by vesicular recruitment. Bladder epithelial cells, for example, display initial unfolding of the apical cell surface and, at later stages, accommodate volume changes by exocytosis of cytoplasmic vesicles^53^.

To illustrate the importance of membrane reservoirs for the mechanical response of cells subject to indentation, we envision the plasma membrane and the underlying cortex as two coupled two-dimensional sheets, each one with its own area modulus ${K}_{{{{{{{{\rm{A}}}}}}}}}^{{{{{{{{\rm{cort}}}}}}}}}$ and ${K}_{{{{{{{{\rm{A}}}}}}}}}^{{{{{{{{\rm{mem}}}}}}}}}$, respectively (Fig. 8b). The parallel arrangement of these two-dimensional “springs” leads to the following expression for the overall tension assuming that the bilayer is purely elastic:

3 $$\sigma (t)={\sigma }_{0}+\int\nolimits_{0}^{t}{K}_{{{{{{{{\rm{A}}}}}}}}}^{{{{{{{{\rm{cort,0}}}}}}}}}{\left(\frac{t-\tau }{{t}_{0}}\right)}^{-\beta }\frac{\partial \alpha (\tau )}{\partial \tau }{{{{{{{\rm{d}}}}}}}}\tau +{K}_{{{{{{{{\rm{A}}}}}}}}}^{{{{{{{{\rm{mem}}}}}}}}}\alpha (t).$$

If we now generate data from equation (3) but assume that the plasma membrane does not participate in the cells’ response to deformation and therefore use equation (1) to fit the data, we find that the fluidity parameter (*β*) becomes smaller as we enlarge the contribution from the membrane (${K}_{{{{{{{{\rm{A}}}}}}}}}^{{{{{{{{\rm{mem}}}}}}}}}$) (Supplementary Note 7 and Supplementary Fig. 9/10/11). This also explains the experimental data from adhered actin-filled GUVs (Fig. 7), where membrane reservoirs are fully exhausted and the response becomes purely elastic as contributions from the in-plane dilatation of the lipid bilayers dominate over cortex extensibility. This can be readily seen by equating the Laplace transforms ($F(s)=L\{f\}(s):= \int\nolimits_{0}^{\infty }{e}^{-st}f(t){{{{{{{\rm{d}}}}}}}}t$) of equations (1) and (3). The apparent fluidity $\widetilde{\beta }$ approaches zero if ${K}_{{{{{{{{\rm{A}}}}}}}}}^{{{{{{{{\rm{mem}}}}}}}}}\gg {K}_{{{{{{{{\rm{A}}}}}}}}}^{{{{{{{{\rm{cort}}}}}}}}}$ and ${K}_{{{{{{{{\rm{A}}}}}}}}}^{0}={K}_{{{{{{{{\rm{A}}}}}}}}}^{{{{{{{{\rm{cort,0}}}}}}}}}+{K}_{{{{{{{{\rm{A}}}}}}}}}^{{{{{{{{\rm{mem}}}}}}}}}$:

4 $$\frac{{K}_{{{{{{{{\rm{A}}}}}}}}}^{0}{{\Gamma }}(1-\widetilde{\beta })}{{s}^{1-\widetilde{\beta }}}=\frac{{K}_{{{{{{{{\rm{A}}}}}}}}}^{{{{{{{{\rm{cort,0}}}}}}}}}{{\Gamma }}(1-\beta )}{{s}^{1-\beta }}+\frac{{K}_{{{{{{{{\rm{A}}}}}}}}}^{{{{{{{{\rm{mem}}}}}}}}}}{s},$$

where ${{\Gamma }}(1-\widetilde{\beta })$ is the Gamma function. The relation results in an apparent logarithmic dependence of $\widetilde{\beta }$ on ${K}_{{{{{{{{\rm{A}}}}}}}}}^{0}$ as exemplarily shown in Fig. 8c. The participation of the membrane in the mechanical response implies that a smaller excess membrane area results in a larger contribution of ${K}_{{{{{{{{\rm{A}}}}}}}}}^{{{{{{{{\rm{mem}}}}}}}}}$ that, in turn, lowers the apparent fluidity of the cell surface, while the fluidity of the cortex remains unaltered. While the linear drop of apparent fluidity seems to be explainable by a loss of excess membrane area and, therefore, a larger contribution of the plasma membrane to the overall response to external deformation, this effect cannot explain that different cells and cells subject to various treatments all lie on such a master curve^54^. However, it might explain the often-observed large standard deviation of viscoelastic parameters.

If the entire excess membrane area is lost, the cells become almost fully elastic and enter a delicate state since already mild triggers such as an osmotic imbalance can lead to membrane rupture and inevitably to cell death. Therefore, it is important that membrane reservoirs remain abundant to protect the cells from lysis. Notably, elevated cortical tension as a response to external stress protects cells from lysis by diminishing in-plane membrane tension.

In conclusion, we investigated the mechanical properties of epithelial cells in response to external cues by targeting the excess surface area. We used a number of different reversible treatments mostly to provoke a decrease in the excess area of cells usually stored in wrinkles, folds, microvilli, endogenous vesicles, and undulations. Lateral stretching of the whole cell monolayer was accomplished with a uniaxial cell stretcher, while cholesterol extraction using a cyclodextrin derivative led to the removal of apical surface structures. Stresses within a cell monolayer are generated by mutants deficient in ZO1/2 displaying a tug-of-war between large, outstretched cells and small, highly contractile cells. Temperature jumps are used to delay the dynamics to provide reservoirs in response to external deformations and to arrest the actomyosin network in a rigor state. Lastly, we also generated GUVs filled with actin filaments to model a reservoir-free cell.

It was found that epithelial cells generally react to dilatation, temperature drop, or removal of cholesterol by quickly sacrificing excess cell surface, i.e., cortex and membrane reservoirs. In response to external cues, the cells also increase their cortical tension and shift the state of the cell towards higher stiffness at preserved fluidity. This allows the cell to change its stiffness independently of fluidity and thereby gain an additional degree of freedom.

It remains, however, to be elucidated how the plasma membrane and the underlying cortex participate in this process. It is clear that the pure presence of a boundary, i.e., the plasma membrane, solidifies the actomyosin cortex and that the additional cross-links between actin and the inner leaflet of the plasma membrane further rigidify the shell of the cell. The attachment sites to the membrane also serve as regulators of cell mechanics through the availability of mediators of membrane actin interactions such as the phosphoinositol PIP_2_ receptors^55^. Importantly, as pointed out by Sens and coworkers, the composite nature of the actin cortex and the plasma membrane implies that the membrane could affect the cytoskeleton for mechanical reasons that are unrelated to biochemical signaling cascades^56^. The membrane/cortex assembly combines a highly elastic but dissipative structure, the actomyosin network, with a passive, almost nonextensible structure, which essentially limits area expansion by its liquid-crystalline nature. Therefore, membrane composition and recruitment of membrane reservoirs are conceivable additional regulators of cell stiffness and can even separate fluidity from stiffness by providing excess area. Here, we could show that exhaustion of membrane reservoirs limits the cell’s ability to function in terms of mechanical adaption processes such as adhesion, migration, and division due to an almost entire loss of fluidity.

## Materials and methods

### Cell culture

The epithelial Madin-Darby Canine Kidney (MDCK II) cell line (ECACC 00062107, strain II, MDCK II; European Collection of Authenticated Cell Cultures, Salisbury, UK) was used for all cell experiments. These were cultured in M10F consisting of minimal essential medium containing Earle’s salts with 2 mM glutaMAX™ (MEM) and 10% fetal bovine serum (FBS, BioWest, France). Cells were incubated in a Heracell 150i (Thermo Fisher Scientific, Waltham, MA, USA) at 37 ^∘^C and a CO_2_ concentration of 5%. Roughly three times per week, cells were subcultured, and the excess cells were used for experiments. Cultivation of the ZO1/2-depleted MDCK II cells is extensively described by Beutel and coworkers^57^. The cells were seeded in Petri dishes and allowed to grow to confluence before measurement.

### Cell stretcher

The expandable measurement chamber was composed of a soft PDMS (polydimethylsiloxane; Slygard 184, Dow Silicones Deutschland, Wiesbaden, Germany) frame (base to cure ratio 25:1) and a 190 μm thin PDMS membrane (10:1) with embedded fluorescent beads to determine the displacement field (see Supplementary Fig. 1/2 and Supplementary Note 1/2). PDMS was degassed before curing. The 25:1 PDMS frames were produced in poly(methyl methacrylate) molds and were cured for 1.5 h at 70 ^∘^C. The PDMS membrane was produced in three steps. First, PDMS (10:1) was spin-coated onto a wafer until a layer thickness of roughly 85 μm is obtained. After curing the PDMS for 45 min at 70 ^∘^C, its surface was activated with 20 s exposure to oxygen plasma. Immediately after activation, 1 ml of a 5 μm in diameter bead solution (0.34 mg ml^−1^, PSI-R5.0K; Kisker Biotech, Steinfurt, Germany) was added and spin-coated. Once dried, a second PDMS layer (10:1) with a thickness of roughly 85 μm was spin-coated on the wafer, and the prior prepared PDMS frames were placed onto the uncured layer. The composite was subsequently cured for 45 min at 70 ^∘^C. After cooling, the PDMS measurement chambers are cut out, placed into Petri dishes, and UV-sterilized (UVLS-26; UVP, Upland, CA, USA) for at least 1 h. The samples were then immediately placed under the sterile laminar flow hood and mounted into the adjustable sample holders. After washing once with PBS, the measurement chambers are coated with Collagen I (bovine, Thermo Fisher Scientific, Waltham, MA, USA) mixed with 0.02 M acetic acid (Carl Roth, Karlsruhe, Germany), resulting in a coating of 5 μg cm^−2^. After a 1 h incubation, the chamber was washed three times with PBS (Biochrom) and once with M10F before seeding 150,000 MDCK II cells in 1.5 ml of M10F. The sample holders were then placed into an incubator (Heracell 150i), where the cells were given time (2 days) growing to confluence.

After confluence was achieved, the sample holders were mounted onto a self-built motorized cell stretcher (see Fig. 1, Supplementary Fig. 1, and Supplementary Note 1). This consisted of a linear stage (M-111.1DG linear stage; Physik Instrumente(PI), Karlsruhe, Germany) which pulled on one side of the sample holder, stretching the PDMS substrate and the cells. Temperature, humidity, and cell media pH (CO_2_ concentration) was controlled with a self-built incubation chamber (see Supplementary Fig. 1 and Supplementary Note 1) and a cage incubator (CellVivo, PeCon, Erbach, Germany).

### Cell patterning

Cultivation and cell patterning was carried out as described by Nehls^30^. The pattern was produced with plasma-induced microcontact printing. Microstructured 5 × 5 mm^2^ PDMS stamps, with a base to cure ratio of 10:1 cured for 4 h at 70 ^∘^C, were produced using passivated microstructured wafers. These stamps were placed into glass-bottom Petri dishes (Ibidi) cleaned with ultrapure water and ethanol. After a 90 s oxygen plasma exposure, a solution of poly-l-lysine-graft-polyethyleneglycol (0.5 ml, PLL (20)-g[3.5]-PEG (2)/tetramethylrhodamine; SuSoS, Dübendorf, Switzerland) was added to each stamp. After a 1 h incubation at 23 ^∘^C, the PDMS stamps were discarded, and the dishes were washed three times with PBS. A Collagen I coating was applied as described above to facilitate cell adhesion. Before seeding with 100,000 MDCK II cells in 2.5 ml M10F with penicillin/streptomycin (0.2 mg ml^−1^; PAA, Pasching, Germany), 0.25 mg ml^−1^ amphotericin B (Biochrom), and 40 mM HEPES (Biochrom)), the dish was washed three times with PBS and twice with MEM. After 60 min of incubation at 37 ^∘^C, the samples were washed to remove nonadherent cells, and the AFM measurements were carried out.

### MBCD treatment

The experimental procedure to remove cellular membrane surface reservoirs is extensively described by refs. ^12,13^. In brief, MDCK II cells were grown to confluence and exposed to MBCD (10 μM; Sigma-Aldrich, Steinheim, Germany) dissolved in M10F and incubated for 1–3 h.

### Atomic force microscopy

NanoWizard 2, 3, 4, or 4 Ultra S atomic force microscopes (AFM; Bruker Nano, Berlin, Germany) mounted on an inverted optical microscope (IX81 or IX83; Olympus, Tokyo, Japan) were used. The temperature was controlled with a Petri dish heater (Bruker Nano, Berlin, Germany) set to 37 ^∘^C. For the cell stretcher experiments, we used an incubation chamber (described above and in Supplementary Note 1 and Supplementary Fig. 1) set to 37 or 25 ^∘^C if required. PDMS measurement chambers were mounted on the cell stretcher in the pre-heated incubation chamber. After an incubation period of at least 30 min, 6–12 cells were indented three times each in short succession using cantilevers equipped with 6.62 μm-sized spherical probes (CP-PNPL-SiO-C, NanoAndMore, Wetzlar, Germany). An approach and retraction velocity of 2 μm s^−1^ with a setpoint of 5 nN were chosen. The strain was applied at a rate of 0.05 mm s^−1^ to the desired motor position, and the measurement position was tracked using the fluorescently labeled beads embedded in the PDMS membrane. For each stretching step, the same cells were indented.

For experiments with MBCD exposure, dKD cells, and actin-filled GUVs MLCT-cantilevers (shape: triangular, nominal spring constant: *k*_nom_ = 0.01 N/m, tip height: *h* = (2.5–8.0) μm, tip radius: *r*_nom_ = 20 nm, Bruker AFM Probes, Camarillo, USA) were used. For cells adhered to ECM patterns we used CSG 11- cantilevers type B (shape: rectangular, nominal spring constant: *k*_nom_ = 0.01–0.08 N/m, tip height: *h* = (2.5–8.0) μm, tip radius: *r*_nom_ = 10 nm; NT-MDT, Zelenograd, Moscow, Russia)

Force curves were obtained at velocities and setpoints shown in Table 1. Only force curves from the center of the cells were used to determine the viscoelastic properties, excluding cell-cell contacts or substrate effects. In some cases, we measured the force relaxation at a constant *z*-piezo position prior to retraction of the cantilever.

**Table 1:** Parameters used for the AFM indentation experiments^13,29,30,35^.

	velocity/μm s^−1^	setpoint/nN
stretcher	2	5
MBCD	2	1
dkD	2	1
cell patterning	5	0.5
GUV	0.5-2.7	2-6

### Actin-filled giant liposomes

The preparation and AFM measurement of giant liposomes (GUVs) filled with actin were carried out as described by refs. ^34,35^. The following lipids were used: 1,2-dioleoyl-*sn-glycero*-3-phospho-choline (DOPC), 1,2-dioleoyl- *sn-glycero*-3-phospho-ethanolamine (DOPE), and 1,2-dioleoyl-*sn- glycero*-3-phospho-ethanolamine-N-(cap biotinyl) (DOPE-Bio) from Avanti Polar Lipids (Alabaster, USA). The ionophore A23187 from Sigma-Aldrich (Steinheim, Germany). The fluorescence marker sulforhod- amine-1,2-dihexanoyl-*sn-glycero*-3-phospho-ethanolamine (TR- DHPE; Life Technology, Carlsbad, USA) was used to label the membranes.

The actin-filled GUVs were produced via electroformation, where 8 μl of a 1 mg ml^−1^ lipid chloroform solution (DOPC/DOPE/A23187/DOPE-Bio/TR-DHPE (59.5:30:5:5:0.5)) were spread over a 12 × 12 mm^2^ area on an indium tin oxide (ITO) slide. In combination with a 1 mm thick square silicone spacer and an additional lipid-covered ITO slide, a seal-able chamber was produced (12 × 12 × 1 mm^3^). To ensure complete solvent removal, the slides were placed in a vacuum oven for at least 3 h at 55 ^∘^C. With 300 μl After complete solvent removal with vacuum for 3 h at 55 ^∘^C, the chamber was filled with 300 μl of a TRIS-HCl buffer consisting of 50 mM sucrose, 2 mM TRIS-HCl, 0.5 mM MgCl_2_, 0.25 mM DTT, 0.20 mM ATP, 5–7 μM actin monomers (rabbit skeletal muscle actin (>95% pure); Cytoskeleton, Denver, USA) and 0.5–1 μM Alexa Fluor 488 actin (labeled rabbit skeletal muscle Alexa Fluor 488; Life Technology) (7.5 pH) and connected to a waveform generator (70 Hz, peak-to-peak 2.4 V) for 3 h at room temperature. The finished GUVs were transferred into a vial for storage at 4 ^∘^C for a maximum of 2 days before being used in experiment^35^.

Experiments were carried out on glass slides which were activated by incubation with NH_4_OH /H_2_O_2_/H_2_O (1:1:5, v/v) for 20 min at 75 ^∘^C. The glass surface was then coated with avidin (1 μM) for 30 min followed by a casein (100 μM) coating for 30 min. After washing with G-buffer (50 mM glucose, 2 mM TRIS-HCl, 0.5 mM MgCl_2_; pH 7.5), 40 μl of the GUV solution was added. After a 10 min incubation, the Mg^2+^ concentration was increased to 2 mM resulting in better GUV fixation and initialization of the actin polymerization^35^.

### AFM imaging

AFM images were obtained in contact mode using sharp MLCT-cantilevers (vide supra). The cells were fixated first with 4% paraformaldehyde (PFA; Fluka, Buchs, Switzerland) in phosphate-buffered saline without Ca^2+^ and Mg^2+^ (PBS; Biochrom), and then after a washing step with PBS, the sample was incubated for 20 min with a glutardialdehyde solution (2.5% (v/v) in PBS). After rinsing with PBS, the samples were stored in PBS, cold (6 ^∘^C) until imaging was carried out.

### Confocal imaging

For membrane staining, cells were incubated with CellMask™ Green (1:500, Life Technologies, Carlsbad, USA) for 5 min prior to fixation with 4% paraformaldehyde (PFA; Fluka, Buchs, Switzerland) in phosphate-buffered saline without Ca^2+^ and Mg^2+^ (PBS; Biochrom) for 20 min at room temperature. Subsequently, the ZO1 junction protein was stained with a mouse AlexFluor 488 monoclonal antibody (339188, Thermo Fisher Scientific, Waltham, MA, USA) for 1 h and rinsed three times with PBS. F-actin was labeled with Alexa Fluor 546 Phalloidin (6.6 μM in PBS, Life Technologies, Carlsbad, USA) for 45 min and rinsed three times with PBS. Nucleus staining was performed by incubation with DAPI (50 ng/μL, Sigma-Aldrich, Steinheim, Germany) for 15 min. Samples were stored in PBS for measurement. Imaging of ZO1/ZO2 depleted MDCK II cells was performed using an inverted confocal laser scanning microscope (CLSM) (FluoView1200; Olympus, Tokyo, Japan) with a 60× oil-immersion objective (UPLSAPO60XO, NA = 1.35; Olympus, Tokyo, Japan). Imaging of cells on PDMS was either carried out with the same CLSM using a 40× objective (UPlanXApo, NA = 0.95; Olympus, Tokyo, Japan), or with an upright CLSM, LSM 900 with Airyscan (Carl Zeiss, Jena, Germany) equipped with a 40× water-immersion objective (40×/1.0 DIC M27 VIS-IR; Carl Zeiss, Jena, Germany).

Imaging of the actin-filled GUVs was carried out with AXIO LSM 710 (Zeiss) using a W Plan Apochromat 63× objective (Zeiss)^35^.

### AFM data evaluation

Data processing and fitting of the AFM time-force curves were carried out with self-written python scripts^58^. Curves were baseline corrected, and the contact point was set in a semi-automated fashion. The fit was then carried out with the help of software packages numba and scipy^59,60^. The resulting viscoelastic properties were merged with cell segmentation data as well as other experimental parameters into large panda data frames for evaluation ^61,62^.

### Cell segmentation

Phase-contrast images of the confluent cell layer were segmented with the software Cellpose^21^. The model *cyto* was used with a flow threshold of 1 and typically a cell probability threshold of −1. When the number of segmented cells was lower than expected, the probability threshold was decreased. These threshold values were kept constant for all the images of each measurement day. With the help of numpy array slicing and the software package skimage, the various cell shape parameters of the segmented cells were determined^22,63^.

### Determining PDMS membrane strain

Fluorescence images prior and during strain were binarized to create large bead clusters, which were then located using opencv^64^. The distance between matched bead clusters was calculated to evaluate the applied strain in strain direction (longitudinal) and perpendicular to it (lateral), respectively.

### Statistics and reproducibility

For statistical analysis a Mann–Whitney–Wilcoxon rank sum test two-sided with Bonferroni correction was used (ns: *p* value > 0.05, *p value < 0.05, **p value < 0.01, ***p value < 0.001, and ****p value < 0.0001). To ensure reproducible strain of the cells, the PDMS composition, curing time, and temperature were kept constant for each experiment (cell stretcher). To reduce cell variation, the seeding number, growth time, and measurement time were kept constant for each experiment. To ensure reproducibility, AFM experiments were performed on different days with different cells.

### Reporting summary

Further information on research design is available in the Nature Research Reporting Summary linked to this article.

## Supplementary information

Supplementary Information

Reporting Summary

## Data Availability

The data that supports the findings of this study are available from the corresponding author upon reasonable request. The underlying numerical data presented in the figures is deposited in GRO.data (Göttingen Research Online) with the following 10.25625/FVI8YF.
