# Supplementary material for: Epithelial cells sacrifice excess area to preserve fluidity in response to external mechanical stress
Source: Commun Biol. 2022 Aug 22;5:855. doi: 10.1038/s42003-022-03809-8 (PMC9395404; doi:10.1038/s42003-022-03809-8)
Supplement: Supplementary file 1 — Supplementary Information [file 42003_2022_3809_MOESM1_ESM.pdf]

Supplementary Information: Epithelial cells sacrifice excess area to preserve fluidity in response to external mechanical stress

Jonathan F. E. Bodenschatz[1], **Karim Ajmail**[1], **Mark Skamrahl**[1], **Marian Vache**[1], **Jannis Gottwald**[1], **Stefan Nehls**[1], **and Andreas Janshoff**[1,*]

[1]Georg-August Universität Göttingen, Institute of Physical Chemistry, Tammannstr. 6, 37077 Göttingen, Germany
[*]ajansho@gwdg.de

Supplementary Note 1: Experimental Setup

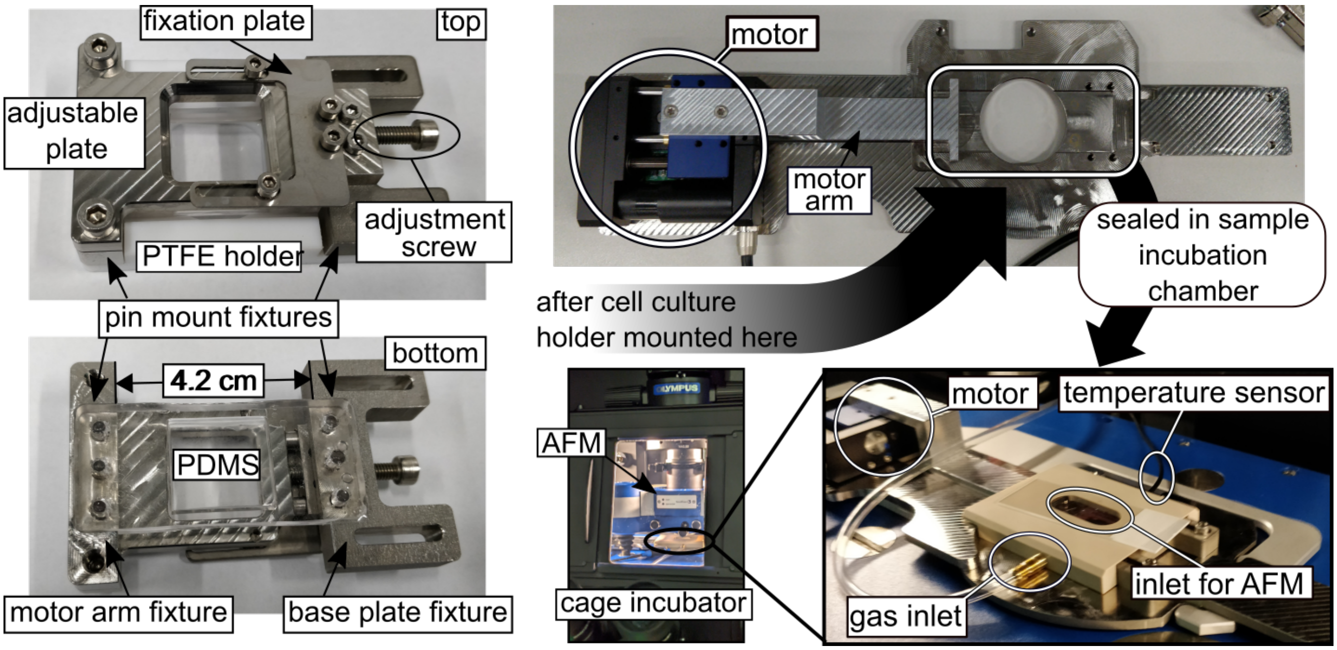

Supplementary Figure 1. Components of the home-built, uniaxial cell stretcher used in this study. On the left the adjustable sample holder in which the PDMS measurement chamber was mounted is displayed. After cells reached the confluent state, the sample holder was attached to the motorized stretcher (upper right). With the adjustable plate removed, the left side was free to move with the motor arm, while the right side was fixed to the base plate. The PDMS chamber is housed in a self-built incubator with an inlet for a saturated air/water with 5% CO_2 mixture, and equipped with a temperature sensor. The incubator was sealed with the AFM during the measurement (lower right). Sample temperature was controlled with a commercial cage incubator from PeCon.

The uniaxial cell stretcher (Supplementary Fig. 1) consists of three main units. (i) An expandable measurement chamber made of polydimethylsiloxane (PDMS; Sylgard 184, Dow Silicones Deutschland, Wiesbaden, Germany), in which the cells were seeded and grown; (ii) an adjustable sample holder, into which the measurement chamber was installed; and (iii) the motorized stretcher (M-111.1DG linear stage; Physik Instrumente(PI), Karlsruhe, Germany), onto which the pre-stretcher was placed. These components were mounted onto an inverted microscope (IX83; Olympus, Tokyo, Japan) equipped with a cage incubator system (CellVivo, PeCon, Erbach, Germany) that contains a self-built sample incubation chamber, allowing for optimal cell culture conditions during the entire measurement. A 40× objective (LUCPlanFLN, NA = 0.60; Olympus, Tokyo, Japan) was used to monitor the confluent layer during the experiment.

Supplementary Note 2: Bead-Based Strain Assessment

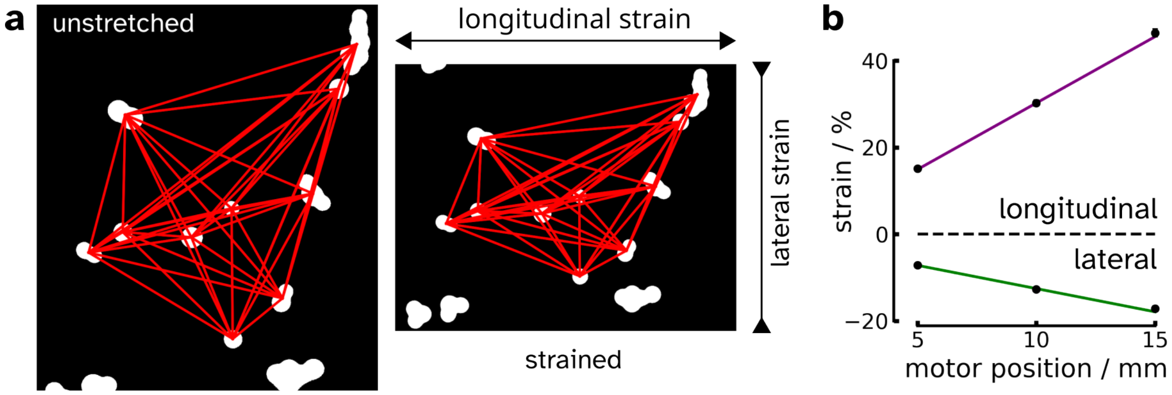

Supplementary Figure 2. Cropped and binarized bead images (**a**) and the resulting strain (**b**). **a** Image of bead-clusters prior to stretching PDMS are shown on the **left** and the beads after application of strain on the **right**, respectively. The red lines denote the distances between the located bead clusters. These are used to calculate the strain in longitudinal and lateral directions. The motor pulls along the longitudinal axis. **b** Measured strain of the PDMS membrane as a function of motor position. In the longitudinal direction the strain is positive and color-coded in purple, while the lateral strain is negative and labelled in green.

To estimate the strain applied to the cells, fluorescently-labeled beads embedded in the PDMS membrane were used. The beads form small clusters that were used to follow the measurement position during strain, and by measuring the change in distances between the bead clusters, the strain of the PDMS membrane can be calculated (Supplementary Fig. 2). Measurements were primarily carried out at the 10 mm motor position resulting in a longitudinal strain (in direction of strain) of 30.23 ± 0.73 % and a lateral strain (perpendicular to strain) of -12.70 ± 0.37 %.

Supplementary Note 3: Cell Segmentation Analysis

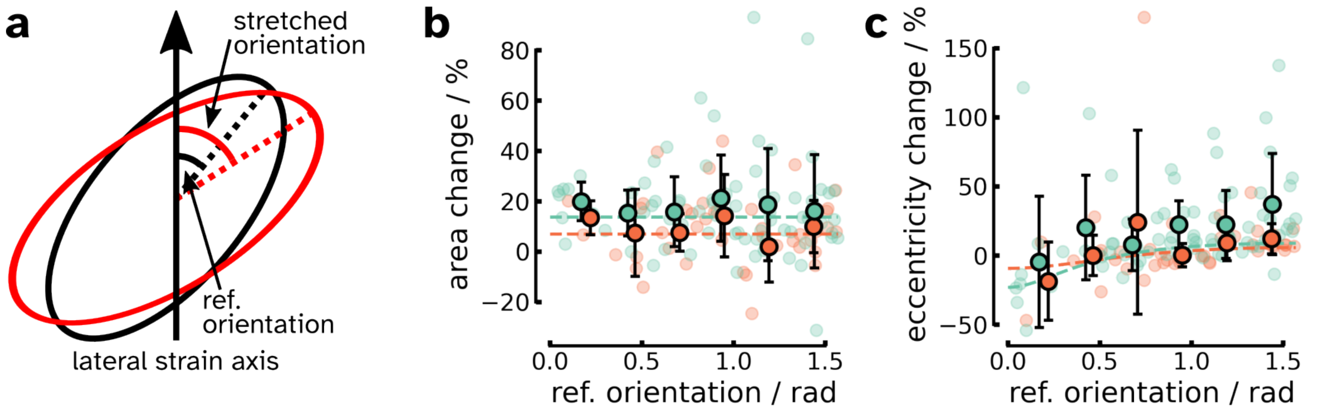

Supplementary Figure 3. Change of cell area and cell eccentricity compared to the expected change. Orange corresponds to approximately 15% axial strain and green to approximately 30% axial strain, respectively. The dashed line is the expected value if the strain of the PDMS-membrane is fully transferred to deform the cells modelled as an ellipse. **a** Eccentricity is evaluated by assuming that the cell's shape can described by an ellipse. The reference orientation and eccentricity were determined before strain was applied. The orientation is defined as the angle between the major axis and the lateral strain axis. During strain, the ellipse is warped, and orientation and eccentricity increase. **b** Area change as a function of orientation angle prior to strain. The calculated area change is shown as a dotted line. Each of the lighter colored points represents a single cell, the points with error bars refer to the binned average with standard deviation, respectively. **c** Eccentricity change as a function of orientation angle prior to strain application. The calculated eccentricity change is shown as a dotted line. Again, lighter colored points are single cells and the points with error bars correspond to the binned average and standard deviation, respectively.

Stretching the cells not only leads to dilation of the projected cell area but also to variations in eccentricity and orientation. The magnitude of these changes are dependent on the cell's orientation before application of strain, whereas the area change is largely independent of the initial orientation (see Fig. 3). The theoretically expected change of the cell area, orientation, and eccentricity were calculated assuming that the measured PDMS strain is fully transferred to the confluent cell layer.

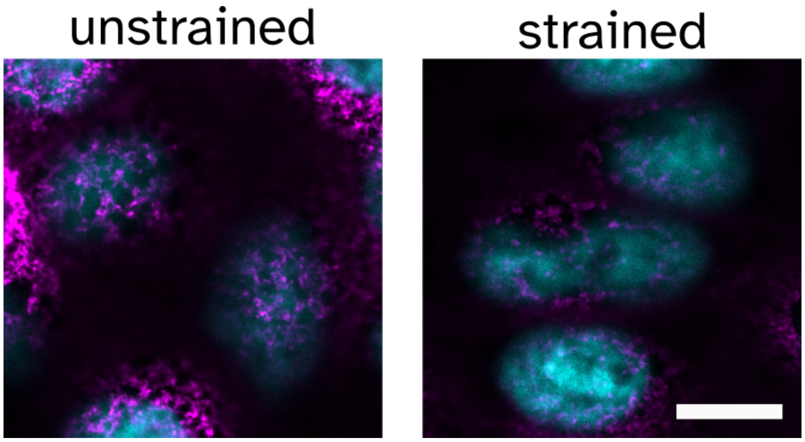

Supplementary Figure 4. CLSM micrographs of immunostained confluent MDCK II cells showing F-actin in magenta and nucleus in cyan (DAPI). The number density of microvilli is reduced after application of lateral strain. Scale bar is 10 μm.

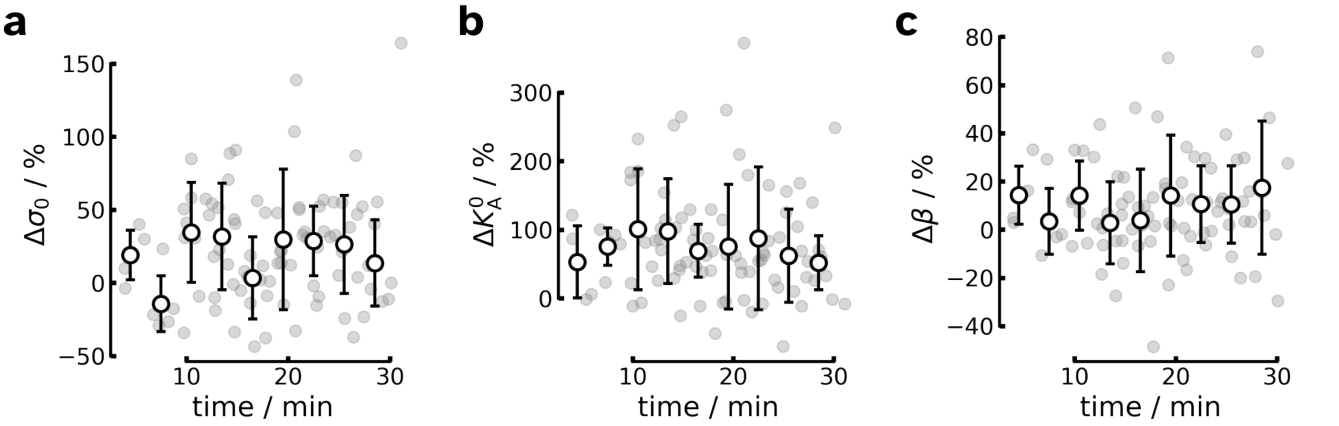

Supplementary Figure 5. Time dependence of the change in pre-stress (cortical tension) $\Delta\sigma$ (**a**), area compressibility modulus (scaling factor) ΔK_A^0 (**b**), and fluidity $\Delta\beta$ (**c**) during the 30 minute period of applied strain. The grey points denote the averaged measurement of each cell, while the large white points represent the binned mean with the standard deviation as the error, respectively. There is no significant adaption of the cells during this time frame.

Supplementary Note 4: Impact of temperature on viscoelasticity

One general way to interfere with the cells' ability to release excess surface area is to lower the temperature and thus slow down the dynamics.[1] For example, assuming that the excess area is recruited from an undulating or corrugated surface, lowering the temperature would render smoothing of the surface more energy costly. This purely mechanical picture must be extended by the temperature dependence of rates governing cytoskeletal dynamics in living cells and by metabolic contributions due to the temperature dependence of enzymes. The idea was to compromise the cell's ability to quickly provide excess area in response to external stimuli by slowing down all processes that might participate in this process.

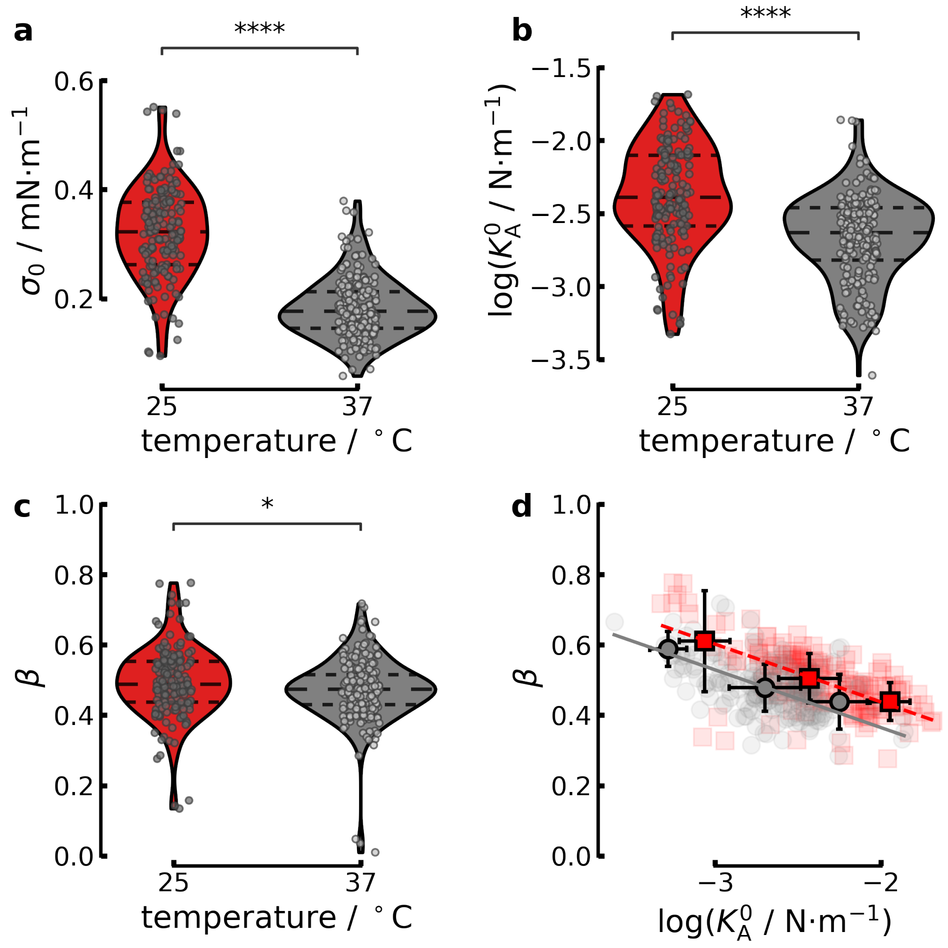

Supplementary Figure 6. Effect of temperature on the viscoelastic properties of MDCK II cells. **a-c** Pre-stress/cortical tension σ_0, the area compressibility modulus K_A^0 and fluidity β. The points are single cells, dark gray for 25 °C (n=57 cells) and light gray for 37 °C (n=67 cells). The dashed lines are the quartiles. **a-b** Both pre-stress/cortical tension σ_0 and the area compressibility modulus K_A^0 increase after decreasing the temperature from 37 °C (gray) to 25 °C (red). **c** Fluidity β remains largely unchanged. **d** Fluidity β as a function of the logarithm of the scaling factor K_A^0. Red (square, dashed lines) refers to 25 °C and gray (circles, continuous lines) to 37 °C. The individual measurements are binned in two dimensions and subject to linear fitting.

We carried out indentation-relaxation experiments at regular culture conditions (37 °C) and at room temperature (25 °C), respectively. Both cortex tension and stiffness increase at lower temperature. Supplementary Fig. 6 shows that the $\beta(\log(K_A^0))$ line shifts indeed to lower cell stiffness at optimal culture conditions, i.e., higher temperatures (37 °C). In living cells, structurally important parameters such as the viscosity of the cytosol, the flow behavior of the cytoskeleton, the polymerization rate of actin, motor activity, binding/unbinding rates of crosslinkers all depend on temperature. While some parameters such as viscosity of the cytosol are instantaneously affected by a change in temperature, a delay can be expected if gene expression or architectural changes are involved. Additionally, we can expect the cell to take appropriate measures in order to reinstall homeostasis upon a change in temperature by triggering multiple cell regulatory interventions further affecting cytoskeletal structure.[2]

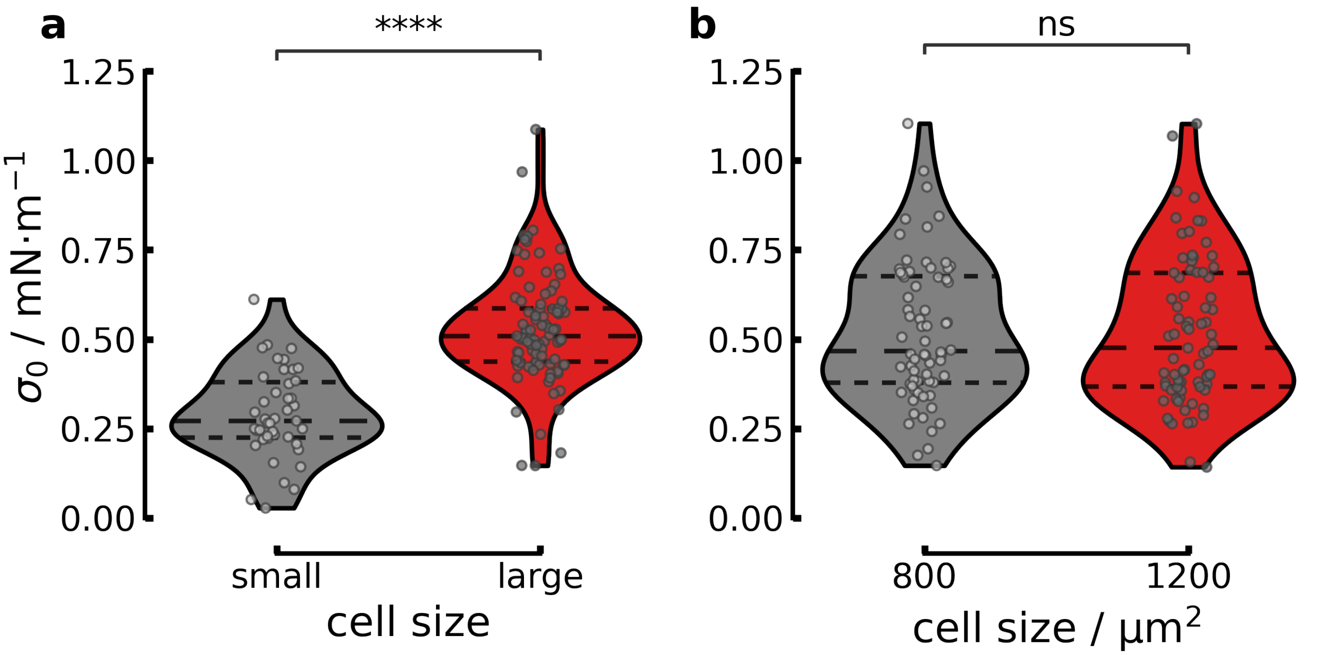

Supplementary Figure 7. Pre-stress/cortical tension σ_0 dependence on cell size of the ZO1/2-depleted MDCK II cells (**a**) and patterned MDCK II cells (**b**), respectively. The points denote single measurements. The thick dashed line is the median, while the thinner dashes are the upper and lower quartile, respectively. **a** Comparison between smaller contractile cells (gray) and larger outstretched cells (red). Data reproduced from Skamrahl et al.[3] **b** No significant difference between the cells grown on the smaller 800 µm^2 large pattern (gray) and cultured on the larger 1200 µm^2 sized pattern (red) is found.

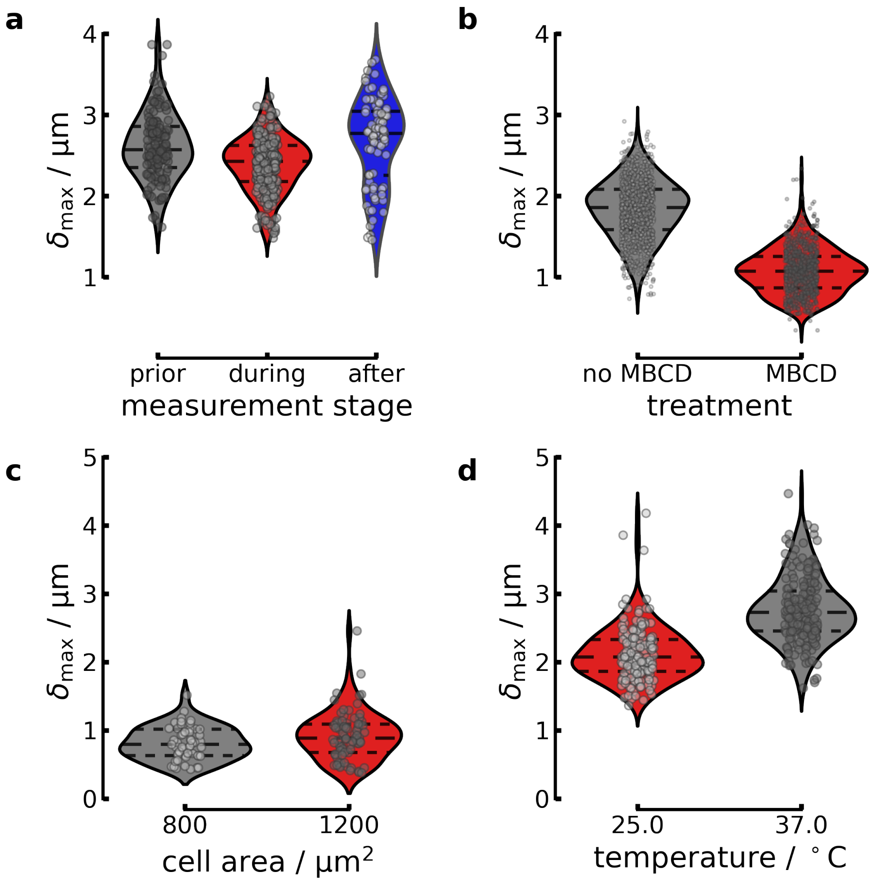

Supplementary Figure 8. The maximum indentation depths δ_{max} reached during the AFM measurements. The points denote single measurements. The thick dashed line is the median, while the thinner dashes are the upper and lower quartile, respectively. **a** Stretcher measurements separated into the different measurement stages. **b** MBCD treatment measurements separated between no MBCD and with MBCD. **c** Cell patterning measurements separated between the extra cellular matrix (ECM) sizes of 800 and 1200 µm^2. **d** Temperature dependence measurements separated between 25 and 37 °C.

Supplementary Note 5: Theory - Force Response of Living Cells

Assuming constant volume of the cells and negligible contributions from bending when deformed by an AFM probe, we only need to compute the minimal surface area to minimize the free energy, as described previously.[4,5] Minimization of the free energy functional $\mathcal{F}$ leads to the well-known Young-Laplace equation, which relates the pressure jump Δp across an interface to the isotropic tension of the shell:[6–8]

$$\frac{\Delta p}{\sigma} = 2H = C_1 + C_2, \tag{1}$$

with σ the tension of the cell surface, the two principle curvatures $C_1 = \frac{1}{R_\theta}$ and $C_2 = \frac{1}{R_\phi}$ defined at each point of the freestanding parts of the cell. The Young Laplace equation implies that surfaces of constant mean curvature are obtained during deformation of the cell. Since cylindrical symmetric problems do not depend on the azimuthal angle ϕ, the Young–Laplace equation reduces to an ordinary differential equation:[6–8]

$$\frac{\Delta p}{\sigma} = \frac{u(r)}{r} + \frac{\mathrm{d}u(r)}{\mathrm{d}r}, \tag{2}$$

where $u(r) = \sin\beta(r)$ and β serves to parameterize the surface as the angle with the surface normal.[9] This relation can easily be integrated to give[6–8]

$$u(r) = Ar + \frac{B}{r}, \tag{3}$$

in which A and B are obtained from appropriate boundary conditions.

Cells with the shape of a spherical cap

Here, we will summarize the treatment if the cells can be described as a spherical cap with a base radius R_1 and a contact angle ϕ_0 deformed with either a conical indenter or a spherical probe. The general procedure has been as detailed previously.[5] Briefly, for a spherical cap that either represents a single adhered cell or the apical cap of a cell that is part of a confluent cell monolayer we obtain for the case of a conical indenter with half cone angle $\theta = \pi/2 - \Theta$:

$$\widetilde{f} = \frac{\sin(\phi) + \widetilde{R}_\mathrm{i}\sin(\Theta)}{1 - \widetilde{R}_\mathrm{i}^2} - \sin\phi = \widetilde{g}^{\mathrm{cone}}_{\mathrm{cap}}(\theta, \phi_0, \widetilde{z}), \tag{4}$$

with ϕ, the contact angle of the cap with the surface changing with $\widetilde{z} = z/R_1$. For a spherical indenter we obtain:

$$\widetilde{f} = \frac{\sin(\phi) + \widetilde{R}_\mathrm{i} \sin\left(\mathrm{atan}\left(\frac{\widetilde{R}_\mathrm{i}}{\sqrt{\widetilde{R}_\mathrm{p}^2 - \widetilde{R}_\mathrm{i}^2}}\right)\right)}{1 - \widetilde{R}_\mathrm{i}^2} - \sin\phi = \widetilde{g}_\mathrm{cap}^\mathrm{sphere}(R_\mathrm{p}, \phi_0, \widetilde{z}), \tag{5}$$

with $\widetilde{R}_\mathrm{p} = R_\mathrm{p}/R_0$, the nondimensional radius of the probe. The generic shape functions $g_\mathrm{sphere}(\widetilde{z})$, $g_\mathrm{cap}^\mathrm{cone}(\theta, \phi_0, \widetilde{z})$ and $g_\mathrm{cap}^\mathrm{sphere}(R_\mathrm{p}, \phi_0, \widetilde{z})$ can be computed once for a given set of parameters ϕ_0, θ and R_p, respectively. The numerically computed shape functions can then be approximated by a polynomial to speed up the computation. Given our initial assumptions, the only source of resistance to deformation originates from the overall tension σ to first order:[7,8]

$$\sigma = \sigma_0 + K_\mathrm{A}\alpha, \tag{6}$$

with σ_0 the time invariant pre-stress of the membrane/cortex shell and K_A, the area compressibility modulus, which reflects the time-dependent elastic resistance to areal strain $\alpha = (\Delta A/A_0)$.[6] Viscoelasticity of this 2D elastic modulus is assumed to obey a power law $K_\mathrm{A} = K_\mathrm{A}^0 \left(\frac{t}{t_0}\right)^{-\beta}$ with $0 \leq \beta \leq 1$ and $t_0 = 1\mathrm{s}$ (set arbitrarily).[4]
Application of the elastic-viscoelastic-correspondence principle leads to the following expression for the overall tension:[4,10]

$$\sigma(t) = \sigma_0 + \int_0^t K_\mathrm{A}^0 \left(\frac{t-\tau}{t_0}\right)^{-\beta} \frac{\partial\alpha(\tau)}{\partial\tau} \mathrm{d}\tau. \tag{7}$$

The hereditary integral(s) can be solved analytically by Laplace Transformation if the corresponding area integrals[4] are also approximated with a polynomial ($\alpha(\widetilde{z}) = \sum_{n=1}^{m} d_n \widetilde{z}^n$).[9] The force response of cells to indentation or compression (approach) at constant velocity ($\widetilde{v}_0 = \widetilde{z}/t$) gives:

$$f_\mathrm{app} = 2\pi\widetilde{g}_\mathrm{cap}(\widetilde{z}) R_1 \left(\sigma_0 + K_\mathrm{A}^0 \sum_n d_n \frac{t^{-\beta} n (\widetilde{v}_0 t)^n \Gamma(1-\beta)\Gamma(n)}{\Gamma(1-\beta+n)}\right). \tag{8}$$

The force response upon retraction at $t = t_\mathrm{m}$, with the identical velocity is:

$$
\begin{aligned}
f_{\text{ret}} =& 2\pi \widetilde{g}_{\text{cap}}(\widetilde{z})R_1 \left(\sigma_0 + K_{\text{A}}^0 \sum_n d_n \left[t^{-\beta} \left(t_{\text{m}}\widetilde{v}_0\right)^n {}_2F_1\left(\beta, n; n+1; \frac{t_{\text{m}}}{t}\right) + \right.\right. \\
& \frac{(-1)^{\beta}\Gamma(\beta+1)\Gamma(n)\left(\frac{1}{2t_{\text{m}}-t}\right)^{-\beta}\left(-\widetilde{v}_0(t-2t_{\text{m}})\right)^n}{\Gamma(\beta+n+1)} - \\
& \left.\left. \frac{(t-t_{\text{m}})^{\beta}\left(\frac{t-t_{\text{m}}}{t-2t_{\text{m}}}\right)^{-\beta}\left(t_{\text{m}}\widetilde{v}_0\right)^n {}_2F_1\left(-\beta, n; n+1; -\frac{t_{\text{m}}}{t-2t_{\text{m}}}\right)}{n}\right]\right),
\end{aligned} \tag{9}
$$

with the Gamma function $\Gamma(n) = \int_0^{\infty} x^{n-1}e^{-x}dx$ and the ordinary hypergeometric function ${}_2F_1(a,b;c;z)$. The corresponding generic shape function, $\widetilde{g}_{\text{cap}}(\widetilde{z})$, needs to be chosen according to the geometry of cell and indenter, respectively. Usually, polynomials to the order of $m = 4$ are sufficient to describe the functions $\widetilde{g}_{\text{cap}}(\widetilde{z},\theta)$ and $\alpha(\widetilde{z},\theta)$ with good accuracy. Experimental force - time curves were subject to fitting a piecewise function $f(t \le t_{\text{m}}) = f_{\text{app}}(t)$ and $f(t > t_{\text{m}}) = f_{\text{ret}}(t)$. In the following, the term cell stiffness refers to the scaling factor of the area compressibility modulus K_{A}^0, while the power law exponent β designates the fluidity of the cell.

Cells or Vesicles with a Spherical Shape

The parametrization of the cell/vesicle with a conical indenter is shown in Fig. 9. Initially, prior to deformation the cell/vesicle assumes an ideal sphere. Starting point for the analysis after indentation is again the Young–Laplace equation with *constant* curvature:

$$
\frac{\Delta P}{T} = C_1 + C_2 = \frac{u(r)}{r} + \frac{\mathrm{d}u(r)}{\mathrm{d}r}. \tag{10}
$$

Integration leads to:

$$
u_i(r) = A_i r + \frac{B_i}{r}, \tag{11}
$$

with $i = 1,2,3$ referring to the corresponding regions of the free contour ($s_1 \rightarrow s_2 (i=1)$, $s_2 \rightarrow s_3 (i=2)$, $s_3 \rightarrow s_4 (i=3)$). For each of the regions, appropriate boundary conditions have to be fulfilled. A_1 and B_1 correspond to the unbound region $i = 1$ ranging from $s_1 \rightarrow s_2$. The following boundary conditions hold:

$$
\begin{aligned}
\beta &= \frac{\pi}{2} \text{ at } r = R_{\text{o}} \\
\beta &= 0 \text{ at } r = R_{\text{i}}.
\end{aligned} \tag{12}
$$

where R_i is the contact radius of the cell/vesicle formed with the flat substrate at the bottom and R_o the equatorial radius of the deformed cell/vesicle (see Supplementary Fig. 9). From Eqs (11, 12) we obtain,

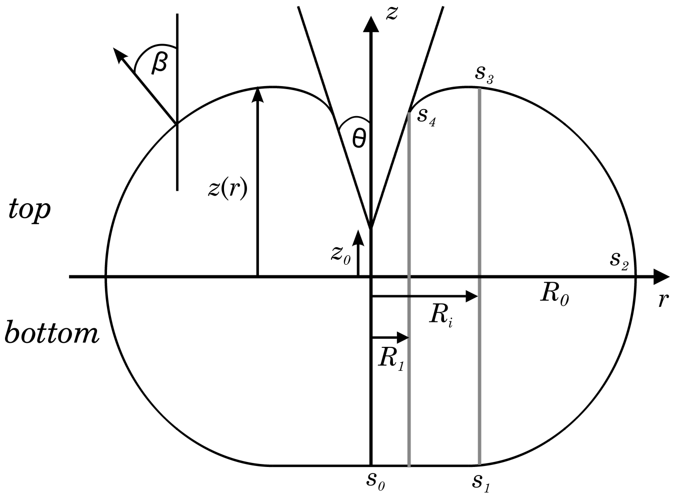

Supplementary Figure 9. Parametrization of a cell/vesicle indented with a conical indenter.

$$A_1 = \frac{R_\mathrm{o}}{R_\mathrm{o}^2 - R_\mathrm{i}^2} \tag{13}$$

$$B_1 = \frac{-R_\mathrm{i}^2 R_\mathrm{o}}{R_\mathrm{o}^2 - R_\mathrm{i}^2} = -A_1 R_\mathrm{i}^2. \tag{14}$$

In region $i = 2$ ($s_2 \rightarrow s_3$) the free contour obeys the boundary conditions:

$$\beta = \frac{\pi}{2} \text{ at } r = R_\mathrm{o} \tag{15}$$

$$\beta = 0 \text{ at } r(s_3). \tag{16}$$

Therefore,

$$A_2 = \frac{R_\mathrm{o}}{R_\mathrm{o}^2 - r(s_3)^2} \tag{17}$$

$$B_2 = \frac{-r(s_3)^2 R_\mathrm{o}}{R_\mathrm{o}^2 - r(s_3)^2} = -A_2 r(s_3)^2. \tag{18}$$

Since the contour is continuous at R_o, $r(s_3) = R_\mathrm{i}$ holds and therefore also $A_1 = A_2$ and $B_1 = B_2$, i.e., the free contour from $s_1 \rightarrow s_2$ and $s_2 \rightarrow s_3$ are mirror-inverted. A_3 and B_3 for region $i = 3$ ($s_3 \rightarrow s_4$) that reaches up to the contact with the indenter at R_1 are obtained from the following boundary conditions:

$$\beta = 0 \text{ at } r = R_\mathrm{i} \tag{19}$$

$$\beta = -\left(\frac{\pi}{2} - \theta\right) \text{ at } r = R_1, \tag{20}$$

leading to

$$
\begin{aligned}
A_3 &= \frac{R_1 \sin\left(\frac{\pi}{2} - \theta\right)}{R_\mathrm{i}^2 - R_1^2} && (21)\\
B_3 &= -A_3 R_1^2 - R_1 \sin\left(\frac{\pi}{2} - \theta\right). && (22)
\end{aligned}
$$

where θ is the half opening angle of the indenter. Once the radii R_o, R_i, and R_1 are found, the free contour corresponding to the regions ($s_1 \to s_2$ using $u_1(r)$, $s_2 \to s_3$ using $u_2(r) = u_1(r)$, and $s_3 \to s_4$ using $u_3(r)$) can be readily obtained from integrating:

$$
\frac{\mathrm{d}z}{\mathrm{d}r} = \tan\beta = \frac{u(r)}{\sqrt{1 - u(r)^2}}. \tag{23}
$$

The remaining contour is defined by the boundaries, a flat substrate at the bottom and the conical indenter from the top.

Now we have to find expressions for R_o, R_1, and R_i depending on the distance between the tip of the indenter and the flat base plate at the bottom z_0. Three conditions apply to an indented cell/vesicle that permit computing force-indentation curves ($f(\delta)$).

1. Volume constraint
The volume of the cell/vesicle prior to indentation is denoted as V_v and the volume of the indented one V_ind. The condition of volume conservation reads:

$$
V_\mathrm{v} = \frac{4}{3}\pi R_\mathrm{v}^3 = V_\mathrm{ind}. \tag{24}
$$

The indented cell/vesicle is a solid of revolution, which facilitates the integration to obtain the volume V_ind:

$$
V_\mathrm{ind} = \int_{R_\mathrm{i}}^{R_\mathrm{o}} \left(\frac{u_1(r)\pi r^2}{\sqrt{1 - u_1(r)^2}} + \frac{u_3(r)\pi r^2}{\sqrt{1 - u_3(r)^2}} \right) \mathrm{d}r - \pi R_\mathrm{i}^2 z(R_i) + \int_{R_1}^{R_\mathrm{i}} \frac{u_3(r)\pi r^2}{\sqrt{1 - u_3(r)^2}} \mathrm{d}r - \frac{\pi R_1^3}{3\tan\theta} \tag{25}
$$

with $z(R_\mathrm{i}) = \int_{R_\mathrm{i}}^{R_\mathrm{o}} \frac{u_1(r)}{\sqrt{1 - u_1(r)^2}} \mathrm{d}r$.

2/3. Force balances
The key assumption is that the only source of the restoring force to indentation is the in–plane tension due to area dilatation. $\Delta A = A_\mathrm{ind} - A_\mathrm{v}$ denotes the difference between the actual area

A_{ind} and the initial area prior to compression A_{v}. The force balance of the top part of the cell/vesicle in the z-direction is:

$$f = 2\pi(R_1 \sin(\pi/2 - \theta) + R_1^2 A_3)\left(T_0 + K_{\text{A}}\frac{A_{\text{ind}} - A_{\text{v}}}{A_{\text{v}}})\right), \tag{26}$$

which is the second condition, while force equilibrium at the bottom part is the third condition:

$$f = \Delta P/\pi R_{\text{i}}^2 = 2\pi R_{\text{i}}^2 A_1 \left(T_0 + K_{\text{A}}\frac{A_{\text{ind}} - A_{\text{v}}}{A_{\text{v}}})\right) \tag{27}$$

The area A_{v} prior to indentation is $4\pi R_{\text{v}}^2$, while the actual area A_{ind} can be divided into two surfaces of revolution, the top $A_{\text{ind}}^{\text{top}}$ and bottom part $A_{\text{ind}}^{\text{bot}}$ of the cell/vesicle according to Fig 9, respectively:

$$A_{\text{ind}}^{\text{bot}} = \pi R_{\text{i}}^2 + 2\pi \int_{R_{\text{i}}}^{R_{\text{o}}} \frac{r}{\sqrt{1 - u_1(r)^2}} \mathrm{d}r \tag{28}$$

$$A_{\text{ind}}^{\text{top}} = 2\pi \int_{R_{\text{i}}}^{R_{\text{o}}} \frac{r}{\sqrt{1 - u_1(r)^2}} \mathrm{d}r + 2\pi \int_{R_1}^{R_{\text{i}}} \frac{r}{\sqrt{1 - u_3(r)^2}} \mathrm{d}r + \frac{\pi R_1^2}{\sin(\theta)}. \tag{29}$$

The indentation depth in the center at $r = 0$ is readily obtained from:

$$\delta = 2R_{\text{v}} - \left(2\int_{R_{\text{i}}}^{R_{\text{o}}} \frac{u_1(r)}{\sqrt{1 - u_1(r)^2}} \mathrm{d}r + \int_{R_1}^{R_{\text{i}}} \frac{u_3(r)}{\sqrt{1 - u_3(r)^2}} \mathrm{d}r - \frac{R_1}{\tan\theta}\right). \tag{30}$$

The contour in region $s_1 \rightarrow s_3$ corresponds to the first integral, while the contour along the path $s_3 \rightarrow s_4$ is represented by the second integral.

The generic shape function j for this geometry reads:

$$\widetilde{f} = \frac{R_{\text{i}}^2}{R_{\text{o}}} A_1 = \frac{R_{\text{i}}^2}{R_{\text{o}}^2 - R_{\text{i}}^2} = \frac{\widetilde{R}_{\text{i}}^2}{1 - \widetilde{R}_{\text{i}}^2} = j(\widetilde{\delta}) \tag{31}$$

with $\widetilde{R}_{\text{i}} = \frac{R_{\text{i}}}{R_{\text{o}}}$. $\widetilde{R}_{\text{i}}$ is computed once and can later be scaled for any radius of the cell/vesicle and the indenter shape, respectively. Again $j(\widetilde{\delta}) = \sum_n a_n \widetilde{\delta}^n$ is approximated with a polynomial to obtain an analytical fitting function. The same is done for the area expansion $\widetilde{\alpha} = \sum_n d_n \widetilde{\delta}^n$. The viscoelastic force response is obtained in the same way as described above (eqn. 8 and 9) just by replacing the corresponding generic shape function g with j.

Supplementary Note 6: Theory - Impact of excess surface area on the measured area compressibility

We refer to the "true" area compressibility modulus, i.e., that which a cortex would display if laterally extended, as $K_\mathrm{A}^\mathrm{true} = A\left(\frac{\partial\sigma}{\partial A}\right)_\mathrm{V,T}$ and to the contribution that comes exclusively from excess area or reservoirs as $K_\mathrm{A}^\mathrm{res}$. Surface reservoirs may comprise thermally excited undulations and more permanent structures depending on cell type or polarity. The two contributions, extensibility of the cortex and recruitment of excess surface area, resist area dilatation as two "springs" arranged in series leading to an apparent area compressibility modulus related to the projected area A_0:[11]

$$K_\mathrm{A} = A_0\left(\frac{\partial\sigma}{\partial A_0}\right)_{V,T}. \tag{32}$$

We assume that we can write $A = A_0 + A_\mathrm{res}$, with A_res the area representing the excess area (in the case of membrane undulations $A \approx A_0 + \frac{1}{2}\iint_{A_0}(\nabla h(x,y))^2\mathrm{d}x\mathrm{d}y$, with $h(x,y)$ the undulating surface) and A_0 the projected area. This allows us to write for the true area compressibility modulus $K_\mathrm{A}^\mathrm{true}$:

$$(K_\mathrm{A}^\mathrm{true})^{-1} = \frac{1}{A}\left(\frac{\partial(A_0 + A_\mathrm{res})}{\partial\sigma}\right)_{V,T} = \frac{1}{A}\left(\frac{\partial A_0}{\partial\sigma}\right)_{V,T} + \frac{1}{A}\left(\frac{\partial A_\mathrm{res}}{\partial\sigma}\right)_{V,T} \tag{33}$$

The first term on the right hand side can be written as $\frac{1}{A}\left(\frac{\partial A_0}{\partial\sigma}\right)_{V,T} = \frac{A_0}{A}(K_\mathrm{A})^{-1}$. If we assume that the recruitment of excess area A_res depends linearly on the external tension σ we arrive at:

$$A_\mathrm{res}(\sigma) = A_\mathrm{res}^0 - \Delta A_\mathrm{res} = A_\mathrm{res}^0 - \frac{\sigma}{K_\mathrm{A}^\mathrm{res}}A, \tag{34}$$

where A_res^0 denotes the initially available excess area, which reduces upon lateral dilatation and $\sigma = K_\mathrm{A}^\mathrm{res}\Delta A_\mathrm{res}/A$. The second term of equation (33) therefore reads:

$$\frac{1}{A}\left(\frac{\partial A_\mathrm{res}}{\partial\sigma}\right)_{V,T} = -\frac{1}{K_\mathrm{A}^\mathrm{res}}. \tag{35}$$

Taken together we obtain for the "true" area compressibility modulus:

$$(K_\mathrm{A}^\mathrm{true})^{-1} = \frac{A_0}{A}(K_\mathrm{A})^{-1} - (K_\mathrm{A}^\mathrm{res})^{-1}, \tag{36}$$

or in terms of the measurable, apparent area compressibility modulus:

$$K_{\mathrm{A}} = A_0 \left(\frac{\partial \sigma}{\partial A_0}\right)_{V,T} = \frac{A_0}{A} \frac{K_{\mathrm{A}}^{\mathrm{true}} K_{\mathrm{A}}^{\mathrm{res}}}{K_{\mathrm{A}}^{\mathrm{true}} + K_{\mathrm{A}}^{\mathrm{res}}} = \xi K_{\mathrm{A}}^{\mathrm{eff}}. \tag{37}$$

Here, the excess area enters through a factor of $\xi = \frac{A_0}{A_0 + A_{\mathrm{res}}}$ into the apparent modulus and thereby modulates the response of cells to deformations.

Supplementary Note 7: Theory - Membrane contribution to cell mechanics

Illustration of the impact of excess membrane on the viscoelastic properties. Assuming a linear ramp in area dilatation (stretching) $\alpha \propto \widetilde{v}_0 t$ we equate:

$$\int_0^t K_{\mathrm{A}} \left(\frac{t-\tau}{t_0}\right)^{-\widetilde{\beta}} \widetilde{v}_0 \mathrm{d}\tau = \int_0^t K_{\mathrm{A}}^{\mathrm{cort}} \left(\frac{t-\tau}{t_0}\right)^{-\beta} \widetilde{v}_0 \mathrm{d}\tau + K_{\mathrm{A}}^{\mathrm{mem}} \widetilde{v}_0 t. \tag{38}$$

At $t = t_0$ we obtain exemplary:

$$\frac{1-\widetilde{\beta}}{K_{\mathrm{A}}} = \frac{1-\beta}{K_{\mathrm{A}}^{\mathrm{cort}} + (1-\beta) K_{\mathrm{A}}^{\mathrm{mem}}}. \tag{39}$$

As demonstrated in figure 10 the scaled stiffness increases due to the presence of a lipid bilayer most pronounced at $\beta = 0.5$.

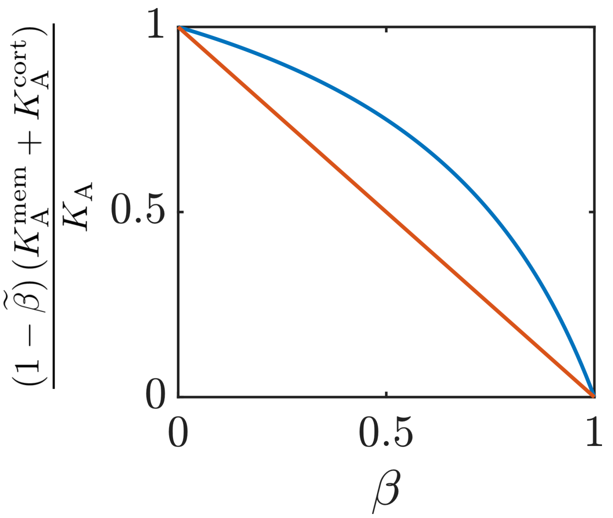

Supplementary Figure 10. Scaled stiffness of cells as a function of actual fluidity of the cortex. The presence of a purely elastic membrane (blue line) contributes to the apparent viscoelastic response of the cortex + membrane by stiffening and reducing apparent fluidity ($K_{\mathrm{A}}^{\mathrm{mem}} / K_{\mathrm{A}}^{\mathrm{cort}} = 2$).

If we now fit force - relaxation curves generated with the right had side of equation (38), i.e. including the presence of membrane in the mechanical response of the shell to deformation, we find a pseudo-stiffening of the cell as illustrated in Supplementary Fig. 11.

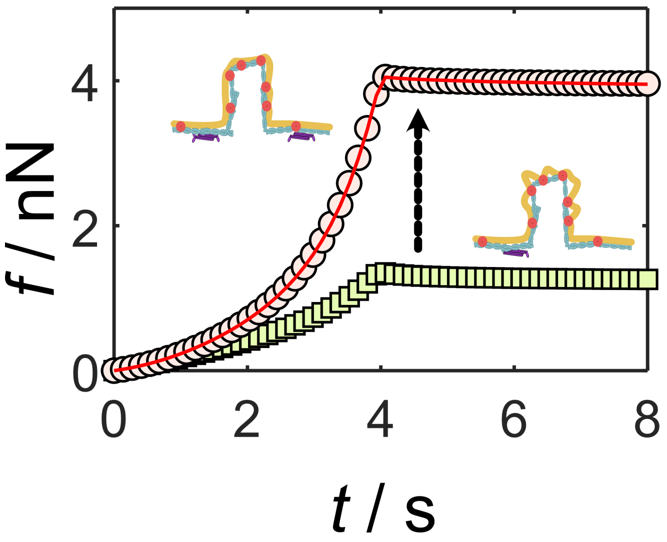

Supplementary Figure 11. Computation of equation (1, rhs) to show the impact of loss of membrane reservoirs on the viscoelastic properties of cells probed with a conical indenter. Compared to a membrane-cortex shell with an infinite amount of membrane reservoirs (squares, $R = 10\,\mu$m, $v = 1\,\mu\text{ms}^{-1}$, $K_A^{\text{mem}} = 0$, $\sigma_0 = 0.3\,$mN/m, $K_A^0 = 0.01\,$N/m, $\xi = 0.1$,$\beta = 0.5$) removal of all existing reservoirs (circles, $K_A^{\text{mem}} = 0.2\,$N/m and all other parameters identical) leads to substantial stiffening of the cell. Fitting the data (red line) with the model that neglects membrane reservoirs leads to $\beta = 0.02$ indicating a purely elastic shell.

Supplementary References

1. Yang, T. *et al.* Investigation of temperature effect on cell mechanics by optofluidic microchips. *Biomed. Opt. Express* **6**, 2991, DOI: 10.1364/boe.6.002991 (2015).

2. Kießling, T. R., Stange, R., Käs, J. A. & Fritsch, A. W. Thermorheology of living cells—impact of temperature variations on cell mechanics. *New J. Phys.* **15**, 045026, DOI: 10.1088/1367-2630/15/4/045026 (2013).

3. Skamrahl, M. *et al.* Tight junction ZO proteins maintain tissue fluidity, ensuring efficient collective cell migration. *Adv. Sci.* **8**, 2100478, DOI: 10.1002/advs.202100478 (2021).

4. Cordes, A. *et al.* Prestress and area compressibility of actin cortices determine the viscoelastic response of living cells. *Phys. Rev. Lett.* **125**, 068101, DOI: 10.1103/PhysRevLett.125.068101 (2020).

5. Nietmann, P. *et al.* Epithelial cells fluidize upon adhesion but display mechanical homeostasis in the adherent state. *Biophys. J.* **121**, 361–373, DOI: 10.1016/j.bpj.2021.12.042 (2022).

6. Evans, E., Waugh, R. & Melnik, L. Elastic area compressibility modulus of red cell membrane. *Biophys. J.* **16**, 585–595, DOI: 10.1016/s0006-3495(76)85713-x (1976).

7. Sen, S., Subramanian, S. & Discher, D. E. Indentation and adhesive probing of a cell membrane with AFM: Theoretical model and experiments. *Biophys. J.* **89**, 3203–3213, DOI: 10.1529/biophysj.105.063826 (2005).

8. Pietuch, A., Brückner, B. R., Fine, T., Mey, I. & Janshoff, A. Elastic properties of cells in the context of confluent cell monolayers: impact of tension and surface area regulation. *Soft Matter* **9**, 11490, DOI: 10.1039/c3sm51610e (2013).

9. Hubrich, H. *et al.* Viscoelasticity of native and artificial actin cortices assessed by nanoindentation experiments. *Nano Lett.* **20**, 6329–6335, DOI: 10.1021/acs.nanolett.0c01769 (2020). PMID: 32786944, https://doi.org/10.1021/acs.nanolett.0c01769.

10. Christensen, R. *Theory of Viscoelasticity* (Elsevier, 1982).

11. Lindahl, E. & Edholm, O. Mesoscopic undulations and thickness fluctuations in lipid bilayers from molecular dynamics simulations. *Biophys. J.* **79**, 426–433, DOI: 10.1016/s0006-3495(00)76304-1 (2000).
