# Supplementary material for: Epithelial cells sacrifice excess area to preserve fluidity in response to external mechanical stress
Source: Commun Biol. 2022 Aug 22;5:855. doi: 10.1038/s42003-022-03809-8 (PMC9395404; doi:10.1038/s42003-022-03809-8)
Supplement: Supplementary file 2 — Reporting Summary [file 42003_2022_3809_MOESM2_ESM.pdf]

nature portfolio

Corresponding author(s): Janshoff, Andreas

Last updated by author(s): Jul 30, 2022

Reporting Summary

Nature Portfolio wishes to improve the reproducibility of the work that we publish. This form provides structure for consistency and transparency in reporting. For further information on Nature Portfolio policies, see our Editorial Policies and the Editorial Policy Checklist.

Statistics

For all statistical analyses, confirm that the following items are present in the figure legend, table legend, main text, or Methods section.

n/a	Confirmed	
☐	☒	The exact sample size (n) for each experimental group/condition, given as a discrete number and unit of measurement
☐	☒	A statement on whether measurements were taken from distinct samples or whether the same sample was measured repeatedly
☐	☒	The statistical test(s) used AND whether they are one- or two-sided<br>*Only common tests should be described solely by name; describe more complex techniques in the Methods section.*
☒	☐	A description of all covariates tested
☒	☐	A description of any assumptions or corrections, such as tests of normality and adjustment for multiple comparisons
☐	☒	A full description of the statistical parameters including central tendency (e.g. means) or other basic estimates (e.g. regression coefficient) AND variation (e.g. standard deviation) or associated estimates of uncertainty (e.g. confidence intervals)
☐	☒	For null hypothesis testing, the test statistic (e.g. F, t, r) with confidence intervals, effect sizes, degrees of freedom and P value noted<br>*Give P values as exact values whenever suitable.*
☒	☐	For Bayesian analysis, information on the choice of priors and Markov chain Monte Carlo settings
☒	☐	For hierarchical and complex designs, identification of the appropriate level for tests and full reporting of outcomes
☒	☐	Estimates of effect sizes (e.g. Cohen's d, Pearson's r), indicating how they were calculated

Our web collection on statistics for biologists contains articles on many of the points above.

Software and code

Policy information about availability of computer code

Data collection	JPK Bruker, Olympus
Data analysis	Python, Matlab, CellPose, Matplotlib, Seaborn, Numpy, Pandas, SciPy, IPython, Numba, Scikit-image, h5py, Statannot

For manuscripts utilizing custom algorithms or software that are central to the research but not yet described in published literature, software must be made available to editors and reviewers. We strongly encourage code deposition in a community repository (e.g. GitHub). See the Nature Portfolio guidelines for submitting code & software for further information.

Data

Policy information about availability of data

All manuscripts must include a data availability statement. This statement should provide the following information, where applicable:

- Accession codes, unique identifiers, or web links for publicly available datasets
- A description of any restrictions on data availability
- For clinical datasets or third party data, please ensure that the statement adheres to our policy

The datasets generated during and/or analysed during the current study are available from the corresponding author on reasonable request.

Field-specific reporting

Please select the one below that is the best fit for your research. If you are not sure, read the appropriate sections before making your selection.

☒ Life sciences ☐ Behavioural & social sciences ☐ Ecological, evolutionary & environmental sciences

For a reference copy of the document with all sections, see nature.com/documents/nr-reporting-summary-flat.pdf

Life sciences study design

All studies must disclose on these points even when the disclosure is negative.

Sample size	Samples size (number of preparations, cell number, number of force curves) was adjusted to reach the anticipated confidence levels.
Data exclusions	No data was excluded from analysis. All individual experiments are displayed in the figures.
Replication	Experiments were repeated with different cells from different days and therefore different preparations as indicated.
Randomization	Cells subject to treatment as indicated and corresponding control cells were randomly selected and subsequently investigated.
Blinding	Data analysis was done with automated scripts that did not distinguish between control and actual experiment. Hence, blinding was not necessary.

Reporting for specific materials, systems and methods

We require information from authors about some types of materials, experimental systems and methods used in many studies. Here, indicate whether each material, system or method listed is relevant to your study. If you are not sure if a list item applies to your research, read the appropriate section before selecting a response.

Materials & experimental systems

n/a	Involved in the study	
☐	☒	Antibodies
☐	☒	Eukaryotic cell lines
☒	☐	Palaeontology and archaeology
☒	☐	Animals and other organisms
☒	☐	Human research participants
☒	☐	Clinical data
☒	☐	Dual use research of concern

Methods

n/a	Involved in the study	
☒	☐	ChIP-seq
☒	☐	Flow cytometry
☒	☐	MRI-based neuroimaging

Antibodies

Antibodies used	ZO1-1A12, AlexaFluor 488, Invitrogen 339188
Validation	ZO1-1A12, This Antibody was verified by Cell treatment to ensure that the antibody binds to the antigen stated. (by manufacturer);

Eukaryotic cell lines

Policy information about cell lines

Cell line source(s)	ECACC 00062107, strain II, MDCK II, European Collection of Authenticated Cell Cultures;<br>MDCK II ZO1/2 dKD provided by Dr. Alf Honigmann, Max Planck Institue of Molecular Cell Biology and Genetics, Dresden
Authentication	None of the cell lines were authenticated.
Mycoplasma contamination	Cells were not tested.
Commonly misidentified lines<br>(See ICLAC register)	None
